# A novel glycosyltransferase catalyses the transfer of glucose to glucosylated anthocyanins in purple sweet potato

**DOI:** 10.1093/jxb/ery305

**Published:** 2018-08-17

**Authors:** Hongxia Wang, Chengyuan Wang, Weijuan Fan, Jun Yang, Ingo Appelhagen, Yinliang Wu, Peng Zhang

**Affiliations:** 1 National Key Laboratory of Plant Molecular Genetics, CAS Center for Excellence in Molecular Plant Sciences, Institute of Plant Physiology and Ecology, Shanghai Institutes for Biological Sciences, Chinese Academy of Science, Shanghai, China; 2 Center for Computational Medicine and Bioinformatics, University of Michigan, Ann Arbor, MI, USA; 3 Shanghai Key Laboratory of Plant Functional Genomics and Resources, Shanghai Chenshan Plant Science Research Center, Chinese Academy of Science, Shanghai Chenshan Botanical Garden, Shanghai, China; 4 John Innes Centre, Norwich Research Park, Colney, Norwich, UK; 5 University of Chinese Academy of Sciences, Beijing, China

**Keywords:** Anthocyanins, glucosyltransferase, glycosyl extension, *Ipomoea batatas*, regioselectivity, stability, UDP-glucose

## Abstract

Glycosylation contributes to the diversity and stability of anthocyanins in plants. The process is catalysed by various glucosyltransferases using different anthocyanidin aglycones and glycosyl donors. In this study, we found that an anthocyanidin 3*-O*-glucoside-2″*-O*-glucosyltransferase (3GGT) from purple sweet potato (*Ipomoea batatas*) catalyses the conversion of anthocyanidin 3*-O*-glucoside into anthocyanidin 3*-O*-sophoroside, which is functionally different from the 3GGT ortholog of Arabidopsis. Phylogenetic analysis indicated regioselectivity of 3GGT using uridine-5′-diphosphate (UDP)-xylose or UDP-glucose as the glycosyl is divergent between Convolvulaceae and Arabidopsis. Homology-based protein modeling and site-directed mutagenesis of Ib3GGT and At3GGT suggested that the Thr-138 of Ib3GGT is a key amino acid residue for UDP-glucose recognition and that it plays a major role in sugar-donor selectivity. Wild-type and *ugt79b1* mutants (defective in UDP carbohydrate-dependent glycosyltransferases, UGTs) of Arabidopsis plants overexpressing *Ib3GGT* produced the new component cyanidin 3*-O*-sophoroside. Moreover, *Ib3GGT* expression was associated with anthocyanin accumulation in different tissues during *I. batatas* plant development and was regulated by the transcription factor IbMYB1. Localization assays for Ib3GGT showed that glycosyl extension occurs in the cytosol and not in the endoplasmic reticulum. This study therefore reveals the function of Ib3GGT in glycosyl extension of anthocyanins and demonstrates that Thr-138 is the key amino acid residue for UDP-glucose recognition.

## Teaser

Sweet potato Ib3GGT catalyses anthocyanidin 3-*O*-glucosides into anthocyanidin 3-*O*-sophorosides using UDP-glucose as the sugar donor, and Thr-138 is the key residue that determines the sugar-donor specificity of the cytosolic enzyme.

## Introduction

Anthocyanins are major secondary metabolites that are responsible for color variation in plants and that exhibit health-promoting properties (de Pascual-Teresa and Sanchez-Ballesta, 2008; He and Giusti, 2010). The basic structures of anthocyanins are mono- and di-glycosylated forms in common anthocyanidins, which include cyanidin, delphinidin, malvidin, pelargonidin, peonidin, and petunidin (Moglia *et al.*, 2014). Different sugar moieties (i.e. glucose, galactose, xylose, arabinose, or fructose) can be linked to hydroxyl groups at the 3, 5, 7, 3′, and 5′ positions, with the glycosylation at the third position on the C-ring being ubiquitous (Andersen and Jordheim, 2010). Glycosylation of 3-OH is catalysed by a series of uridine-5′-diphosphate (UDP) carbohydrate-dependent glycosyltransferases (UGTs), which utilize the nucleotide-activated sugars as donor substrates and anthocyanidin aglycones or anthocyanins as acceptors. These activities increase the structural diversity of anthocyanins by the addition of different types and/or numbers of sugar moieties on various positions (Gachon *et al.*, 2005). The glycosylation of anthocyanin is speculated to occur on the cytoplasmic surface of the endoplasmic reticulum (ER), and may serve as a signal in regulation of the transport of anthocyanins to vacuoles via multiple pathways; this transport is essential for the stable storage of anthocyanins in vacuoles (Ono *et al.*, 2006; Matsuba *et al.*, 2010; Zhao *et al.*, 2011, 2015; Sun *et al.*, 2012). Glycosylation also participates in the fine adjustment and stabilization of flower pigmentation in ornamental plants (Yonekura-Sakakibara *et al.*, 2012).

Monoglycosylation of anthocyanidins produces anthocyanidin 3-*O*-glucosides, the first major stable colored pigment in the anthocyanin biosynthesis pathway (Griesser *et al.*, 2008*a*; Montefiori *et al.*, 2011). Deficiency of activity of the corresponding UDP-glucose:flavonoid 3*-O*-glycosyltransferase (UF3GT), in maize *bronze1* and Arabidopsis *anl1* results in a significantly suppressed accumulation of anthocyanin (Fedoroff *et al.*, 1984; Kubo *et al.*, 2007). UF3GT has been well-characterized as a UGT related to anthocyanin biosynthesis (Gachon *et al.*, 2005; Yonekura-Sakakibara and Hanada, 2011). Glycosyl extension of anthocyanidin 3*-O*-glucosides involves diverse sugars across different species, such as UDP-rhamnose, UDP-glucose, UDP-xylose, and UDP-arabinose, which function as donor substrates added at species-specific positions to the glycosides of mono 3-*O*-glycosylated anthocyanins (Yonekura-Sakakibara *et al.*, 2012). UGT mutants affected in this glycosyl extension function may show effects in anthocyanin accumulation, as reported in petunia and Japanese morning glory (Kroon *et al.*, 1994; Morita *et al.*, 2005). Because all the UGT proteins are highly similar in their secondary and tertiary structures, with a defined fold-structure and highly conserved putative secondary product glycosyltransferase (PSPG) motifs (Breton *et al.*, 2006; Lairson *et al.*, 2008; Osmani *et al.*, 2009), structure-based modeling has been able to identify the key residues of UF3GT responsible for sugar-donor specificity (Kubo *et al.*, 2004) in Arabidopsis (Kim *et al.*, 2013), *Freesia* hybrids (Sun *et al.*, 2016), grapes (Offen *et al.*, 2006; Ono *et al.*, 2010), Lamiales and *Perilla* (Noguchi *et al.*, 2009), and red daisy (Osmani *et al.*, 2009).

Although the anthocyanidin modification by glycosylation is progressive, it commonly begins with 3-*O*-glycosylation, which ensures the stability of the aglycon. Additional glycosylation leads to diversity of compounds and functions, thereby contributing to the variety of anthocyanins in plants (Gachon *et al.*, 2005; Caputi *et al.*, 2012). To date, more than 600 anthocyanins or their derivatives have been identified in nature (Glover and Martin, 2012); however, only a limited number of genes encoding UFGTs in different species are well characterized. Several flavonoid 3*-O*-glycosyltransferases have been characterized in Arabidopsis (Kubo *et al.*, 2007; Saito *et al.*, 2013), strawberry (Griesser *et al.*, 2008*a*, 2008*b*), grapes (Offen *et al.*, 2006), and maize (Fedoroff *et al.*, 1984). In addition, UGTs with further multiple flavonoid glycosylation have also been characterized, including anthocyanidin 3*-O*-glucoside 6″*-O*-rhamnosyltransferase in *Petunia hybrida* (Kroon *et al.*, 1994), anthocyanidin 3*-O*-glucoside 2″*-O*-glucuronosyltransferase in red daisy flowers (Sawada *et al.*, 2005), and flavonol 3*-O*-glucoside 2″*-O*-glucosyltransferase in Arabidopsis (Yonekura-Sakakibara *et al.*, 2014). The divergence towards different glycosylation types occurs at this step. At the same 2″ position, different glycosylation types (i.e. glycosylation or xylosylation) are found in various plant species. In morning glory, anthocyanidin 3*-O*-glucoside 2″*-O*-glucosyltransferase catalyses the addition of a glucose molecule to anthocyanidin 3*-O*-glucosides on the 2″ position to form anthocyanidin 3*-O*-sophorosides (Morita *et al.*, 2005). In Arabidopsis, glycosyl extension of the 3-*O*-glucoside is catalysed by anthocyanidin 3*-O*-glucoside 2″*-O*-xylosyltransferase (AtA3G2XylT, i.e. At3GGT), which adds one xylose molecule specifically to the first glucose residue (Yonekura-Sakakibara *et al.*, 2012). Further modifications, for example malonylation and aromatic acylation, rely on the glycosylation of anthocyanidins to extend their diversity or functionality (Sasaki *et al.*, 2014).

Purple sweet potato (*Ipomoea batatas*) accumulates large amounts of anthocyanins in storage roots, with anthocyanidin 3*-O*-glucoside-2″*-O*-glucoside (anthocyanin 3*-O*-sophoroside) and derivatives being the major compounds (Tian *et al.*, 2005). To date, at least 26 components have been identified, mostly caffeoylated, coumarylated, or feruloylated anthocyanidin glucosides (Truong *et al.*, 2010; Lee *et al.*, 2013). This compares with only 11 anthocyanins that have been identified in Arabidopsis, all of which are derived from cyanidin 3*-O*-glucoside-2″*-O*-xyloside (Tohge *et al.*, 2005; Yonekura-Sakakibara *et al.*, 2012; Kovinich *et al.*, 2014). Thus, unlike Arabidopsis, purple sweet potato uses UDP-glucose as the sugar donor for glycosyl extension of anthocyanidin 3*-O*-glucosides to form anthocyanidin 3*-O*-sophorosides. In the present study, we characterized a UFGT, termed as UDP-glucose:anthocyanidin 3*-O*-glucoside-2″*-O*-glucosyltransferase (IbA3G2GluT, i.e. Ib3GGT) that catalyses the anthocyanin glycosylation in purple sweet potato and identified its key amino acid for sugar-donor selectivity.

## Materials and methods

### Plant materials

The purple-ﬂeshed sweet potato (*Ipomoea batatas* Lam.) cultivar Ayamurasaki was used in this study. *In vitro* shoot cultures were subcultured on SBM medium (Murashige and Skoog salts including vitamins + 0.3 mg l^–1^ vitamin B1 + 30 g l^–1^ sucrose, pH 5.8) in plant growth chambers under a 16-h photoperiod provided by cool-white fluorescent tubes (∼50 μmol m^–2^ s^–1^), at 25 °C and 50% relative humidity. Plantlets at 1 month old were transplanted into plastic pots containing well-mixed soil (soil:peat:perlite, 1:1:1) and grown in a greenhouse (16/8 h light/dark cycle, 25 °C day/night). One-month-old pot-grown plants were transplanted to the field at the Wushe experimental station, Songjiang, Shanghai in early May for evaluation of phenotypes and agronomic traits. These plants were grown for 5 months under standard cultivation conditions. Samples of leaves, stems, fibrous roots, and storage roots were taken at different developmental stages from the pot- and field-grown plants for multiple analyses. Arabidopsis plants were grown under a 16/8 h light/dark cycle, at 22 °C in a growth chamber.

### Plasmid construction and production of transgenic sweet potato

The ORF of *Ib3GGT* (1380 bp) was amplified from the cDNA of sweet potato cv. Ayamurasaki using the primers Ib3GGTF (5′-CGG**GGTACC** ATGGGTTCTCAAGCAACAAC-3′, KpnI site in bold) and Ib3GGTR (5′-AAT**GTCGAC**TCATCCAAGGAGATCCTGCA-3’, SalI site in bold). This fragment was inserted into the KpnI/SalI sites of the pCAMBIA1301-based plant expression vector to generate the binary vector pOE-Ib3GGT containing the expression cassette of *Ib3GGT* driven by the CaMV 35S promoter. The pRNAi-Ib3GGT binary vector was manipulated to express double-stranded hairpin RNA of the 252-bp *Ib3GGT* fragment (382–633 bp) based on the pRNAi-DFR vector (Wang *et al.*, 2013). Then, pOE-Ib3GGT and pRNAi-Ib3GGT were introduced into *Agrobacterium tumefaciens* strain LBA4404 for transformation of sweet potato, as described previously (Yang *et al.*, 2011). Transgenic plants were produced and verified for *Ib3GGT* expression by real-time RT-PCR. For total *Ib3GGT* expression, an internal primer pair of *Ib3GGT* was designed for detecting the *Ib3GGT* expression in the wild-type (WT), Ib3GGT-OE (over-expressing), and Ib3GGT-RNAi plants by real-time RT-PCR (Supplementary Table S2 at *JXB* online). The *Actin* gene of sweet potato was used as an internal control for gene amplification.

### Transformation and analysis of *Ib3GGT*-overexpressing Arabidopsis

Two independent UGT79B1 Arabidopsis transposon mutants, *ugt79b1-1* and *ugt79b1-2* (Kuromori *et al.*, 2004; Ito *et al.*, 2005), together with the WT Nossen and ecotype Col-0 were transformed with *A. tumefaciens* LB4404 harboring pOE-Ib3GGT, using the floral dip method (Clough and Bent, 1998). The transformants were selected on half-strength Murashige and Skoog medium containing 50 mg l^–1^ hygromycin for Nossen and the mutants, or 25 mg l^–1^ hygromycin for Col-0 plants. RNA extracted from T3 homozygous Arabidopsis seedlings was used for RT-PCR analysis. The primer pairs used to detect the expression of *At3GGT* and *Ib3GGT* in the WT and transgenic plants were designed using the software Primer 3.0 and are listed in Supplementary Table S2. *At3GGT* was amplified with a 223-bp fragment from position +369 to +591 bp and *Ib3GGT* was amplified with a 189-bp fragment from +1009 to +1197 bp. The *Actin* gene of Arabidopsis was used as the reference.

### Phylogenetic analysis

To construct a phylogenetic tree, 16 UGT protein sequences obtained from NCBI GenBank were aligned using ClustalW and implemented in MEGA6 (Tamura *et al.*, 2013). Ten closely related UGTs were used to illustrate the relationship. The maximum likelihood method was used to obtain the alignment results (Stamatakis, 2014). Bootstrap values were obtained with 1000 replications.

### Site-directed mutagenesis and *in vitro* enzymatic assays of recombinant Ib3GGT and At3GGT

The full-length sequence of the *Ib3GGT* gene was amplified by PCR using the primers IbGGT-FP (5′-CCC**AAGCTT** ATGGGTTCTCAAGCAACAAC-3′, HindIII site in bold) and IbGGT-RP (5′-CGC*GG***ATCC**TCA**CATCACCATCACCATCAC**TCCAAGG AGATCCTGCA-3′’, BamHI site and 6 His sites in bold). The full-length *At3GGT* was amplified by PCR using the primers AtGGT-FP (5′-GG**GGTACC**ATGGGTGTTTTTGGATCGAA-3′, KpnI site in bold) and AtGGT-RP (5′-CG**GAATTC**TCA**CATCACCATCACCATCAC** TGACTTCACAAGTTCAATTAAATT-3′, EcoRI site and 6 His sites in bold). Site-directed mutations were generated by changing the Thr-138 nucleotide ACC into ATT in *Ib3GGT* and Ile-142 ATC into ACT in *At3GGT* using PCR-based amplification with a Phusion Site-Directed Mutagenesis Kit (Thermo Scientific). The sequence fragments, with or without the mutation of the 3GGTs, were cloned into the pYES2 vector and introduced in *Saccharomyces cerevisiae* BY4742 according to the manufacturer’s instructions (Invitrogen. Cat. no. V825-20). The recombinant 3GGT proteins were induced by replacing the carbon source from 2% glucose to 2% galactose in the SC-U medium. The reaction mixture for the 3GGT enzymatic assay consisted of 100 mM phosphate buffer (pH 7.0), 0.6 mM flavonoid aglycones (cyanidin, cyanidin3*-O*-glucoside, cyanidin 3,5*-O*-diglucoside, or flavonol 3*-O*-glucoside), 1 mM UDP-glucose, and 20 μl of crude yeast extract as the enzymatic solution in a reaction volume of 100 μl. After incubation for 2 h at 37 °C, the reaction was terminated by centrifugation. The enzymatic activity of mutant 3GGT was assessed by cyanidin 3-*O*-glucoside as the acceptor substrate and different UDP-sugars (UDP-glucose, UDP-xylose, UDP-galactose, or UDP-arabinose) as the sugar donor.

### LC-MS analyses of metabolites obtained by enzymatic reaction

Samples of 10 µl of filtered supernatants were analysed on a HPLC1200-MSD/Q-TOF 6520 system (Agilent) as described previously (Wang *et al.*, 2013). Briefly, the mobile phase consisted of 0.5% (v/v) acetic acid in water (eluent A) and 100% acetonitrile (eluent B). Samples were eluted at a flow rate of 0.2 ml min^–1^ and passed through a reverse-phase C18 column (Agilent ZORBAX Eclipse XDB, 4.6 × 50 mm, ID 1.8 μm), and anthocyanin was monitored using a DAD detector at 530 nm. Subsequently, an ESI interfaced Q-TOF mass detector (*m/z* 40–1500) collected the mass *m*/*z* data, which were processed using Agilent Mass Hunter Qualitative Analysis (version 3.0) in order to obtain accurate estimations of molecular mass and to evaluate the spectra. Cyanidin 3-*O*-sophoroside (Tongtian, Shanghai, China) was used as a standard.

### Subcellular localization of Ib3GGT in plant cells

The *Ib3GGT* gene was amplified by PCR using Pfu polymerase (Takara) to obtain a non-stop coding sequence using the primers FPGGT_L (5′-AAT**GTCGAC**ATGGGTTCTCAAGCAACAAC-3′, SalI site in bold) and RPGGT_L (5′-GG**ACTAGT**CCAAGGAGATCCTGCAGTT-3′, SpeI site in bold). Ib3GGT-eGFP was constructed by inserting the *Ib3GGT* fragment into the corresponding sites of a modified pCambia1300 to fuse with the enhanced green fluorescent protein (eGFP) coding sequence. The construction of the ER-marker (Nelson *et al.*, 2007) and the expression construct for monomeric red fluorescent protein (mRFP; Claudia *et al.*, 2017) have been described elsewhere. The ER marker, ER-mCherry, contains a signal peptide of AtWAK2 at the N-terminal and a synthetic HDEL at the C-terminal (He *et al.*, 1999; Nelson *et al.*, 2007). All constructs were introduced into *A. tumefaciens* GV3101 (pMP90). The growth conditions for *Nicotiana benthamiana* and *A. tumefaciens*, as well as the agro-infiltration procedure, have been described previously (Leuzinger *et al.*, 2013). Images were acquired 36 h post-infiltration using a Leica SP8X confocal microscope equipped with a Leica HC PL APO CS2 63×/1.20 water immersion objective. GFP fluorescence was detected by a hybrid detector HyD1 in the range of 500–540 nm and excited using the 488-nm line of an argon ion laser. mCherry and mRFP fluorescence were detected in the range of 580–630 nm by HyD2 after excitation at 561 nm with a diode-pumped solid-state laser. Both fluorophores were recorded line-by-line sequentially at a 3- to 4-fold average in a background noise-dependent manner. The Leica Application Suite X software was used for image acquisition and estimations of intensity.

### Anthocyanin measurement and detection

Total anthocyanins in the WT and transgenic lines were extracted using previously described methods with slight modifications (Wang *et al.*, 2013). The total contents of anthocyanin in the WT and transgenic lines were quantified as cyanidin 3-*O*-sophoroside equivalents. Anthocyanin autofluorescence in epidermal cells of sweet potato leaves was examined using a PCM-2000/Nikon Eclipse 600 laser-scanning microscope (Nikon, Japan) equipped with an argon and helium-neon laser (excitation 488 nm, emission 544 nm).

### Luciferase assays

The *Ib3GGT* promoter (2000 bp) was amplified by the primers Ib3GGTprFP (3′-AAC**TGCAG**TTCAGTCAGGCAATCACAGG-5′, PstI site in bold) and Ib3GGTprRP (3′-CGC**GGATC** CAATAATACCTAGCTAGCT-5′, BamHI site in bold) and cloned into the pLL00R vector to generate the luciferase (LUC) reporter vector. The *IbMYB1* gene was amplified by the primers IbMYB1FP (3′-GGG**GTACC**ATGGTTATTTCATCTGTATG-5′, KpnI site in bold) and IbMYB1RP (3′-AAC**TGCAG**TTAGCTTAACAGTTCTGAC-5′, PstI site in bold) and subcloned into pCAMBIA1300 to generate the CaMV35S-IbMYB1 effector plasmid. For assessing the luciferase activity, *A. tumefaciens* strain GV3101 harboring the *Ib3GGT* promoter–LUC reporter and CaMV 35S-IbMYB1 effector was infiltrated into 5-week-old *N. benthamiana* leaves using a needleless syringe. The plants were grown for 48 h (16/8 h light/dark cycle, 25 °C day/night), after which the leaves were injected with 0.94 mM luciferin as the substrate. The leaves were collected in the dark after 3 min and luciferase signals were detected on a Tanon-5200 image system. The LUC reporter empty vector with 35S-IbMYB1 or *Ib3GGT* promoter–LUC reporter with an empty effector vector was also co-infiltrated as a negative control. These experiments were repeated at least three times, and similar results were obtained.

### Molecular modeling of Ib3GGT and At3GGT active sites

3D models of Ib3GGT and At3GGT were generated using the SWISS-MODEL workspace (Biasini *et al.*, 2014; Wetterhorn *et al.*, 2016) and the I-TASSER server (Yang *et al.*, 2015) based on the structure of *N*-/*O*-glucosyltransferase of *A. thaliana*, which served as a template [UGT72B1 Protein Data Bank (PDB) ID: 2VCE; Brazier-Hicks *et al.*, 2007]. The substrate-binding sites were predicted by superposing both models to UGT72B1 using the *Coot* program (Emsley *et al.*, 2010).

### Statistical analyses

All data were represented as means (±SD) from at least three biological replicates. One-way ANOVAs were performed using SPSS Statistics 17.0 for Duncan’s multiple comparison tests.

## Results

### Comparison of anthocyanins indicates different glycosyl extension patterns in sweet potato and Arabidopsis

In purple sweet potato cv. Ayamurasaki, anthocyanins include aromatically acylated anthocyanidin 3*-O*-sophoroside and derivatives, whereas in Arabidopsis Col-0, the anthocyanin components are anthocyanidin 3*-O*-glucoside-2″*-O*-xylosyl derivatives (Tian *et al.*, 2005; Tohge *et al.*, 2005; Supplementary Table S1). This implies that, although the first glycosylation step of anthocyanins is similar, further modifications of anthocyanidin 3*-O*-glucosides diverge based on the utilization of different sugar donors: in Arabidopsis glycosyltransferase UGT79B1 (At3GGT) catalyses the conversion of UDP-xylose and cyanidin 3*-O*-glucoside, and in sweet potato we predict that UDP-glucose:anthocyanidin 3*-O*-glucoside-2*-O*-glucosyltransferase (Ib3GGT) participates in glycosyl extension (Tohge *et al.*, 2005; Saito *et al.*, 2013).

### Cloning and phylogenetic characterization of Ib3GGT

**Fig. 1.**
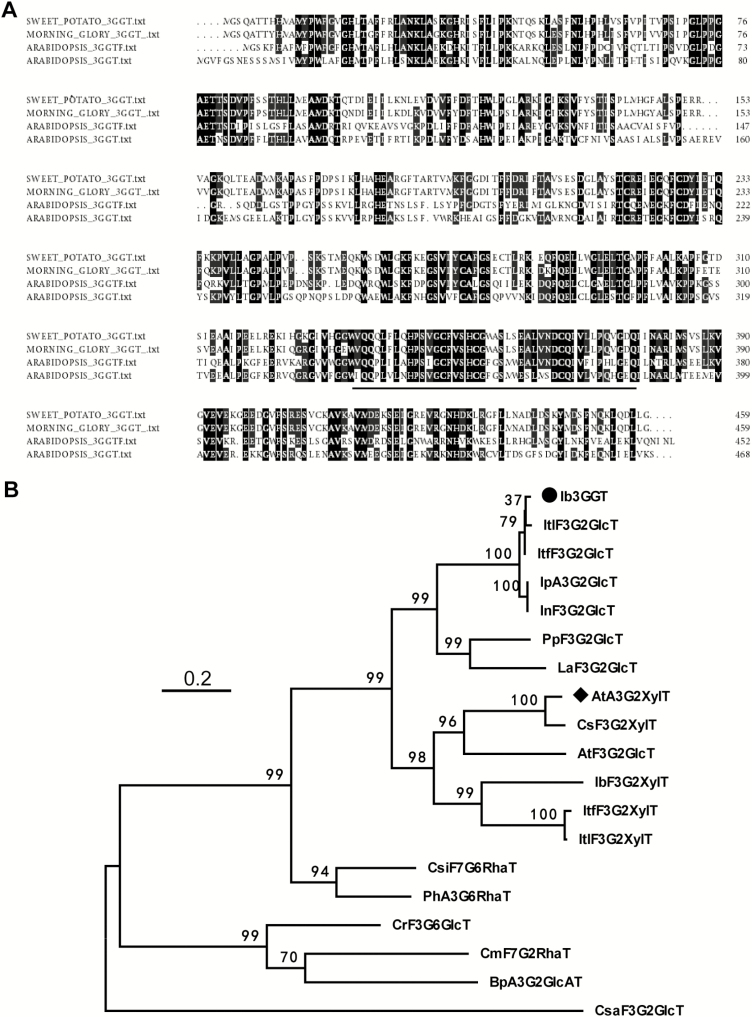
Alignment of amino acid sequences and phylogenetic tree of flavonoid glycosyltransferases. (A) Multiple alignments of amino acid sequences of sweet potato Ib3GGT, morning glory Ip3GGT, and Arabidopsis At3GGTF and At3GGT. The underlined nucleotides represent the putative C-terminal UDP-binding motif for putative secondary product glycosyltransferases (PSPG). (B) Non-rooted molecular phylogenetic tree of flavonoid glycosyltransferases from selected plant UDP-glycosyltransferases. All amino acids were aligned using CLUSTALW. Bootstrap values from 100 retrials are indicated at each branch. The scale shows 0.2 amino acid substitutions per site. Abbreviations for species: Ac, *Actinidia chinensis*; At, *Arabidopsis thaliana*; Bp, *Bellis perennis*; Cm, *Citrus maxima*; Cr, *Catharanthus roseus*; Cs, *Camelina sativa*; Csa, *Crocus sativus*; Csi, *Citrus sinensis*; Ib, *Ipomoea batatas*; In, *Ipomoea nil*; Ip, *Ipomoea purpurea*; Itf, *Ipomoea trifida*; Itl, *Ipomoea triloba*; La, *Lupinus angustifolius*; Ph, *Petunia hybrida*; Pp, *Prunus persica*. The GenBank accession numbers or genome sequence codes for the sequences are as follows (in parentheses): AtA3G2″XylT (NP_200217); AtF3G2″GlcT (NP_200212); BpA3G2″GlcAT (AB190262); CmF7G2″RhaT (AY048882); CrF3G6″GlcT (BAH80312); CsF3G2″XylT(XP_018450414); CsaF3G2″GlcT(CCG85331); CsiF7G6″RhaT (NP_001275829); Ib3GGT (ABL74480); IbF3G2″XylT (XP_019151635); IpA3G2″GlcT (AB192315); InF3G2″GlcT (XP_019194233); ItF3G2″GlcT (itf02g12970.t1); ItF3G2″GlcT (itb02g08330.t1); ItF3G2″XylT (itb03g28310.t1); ItF3G2″XylT (itf03g22690.t2); LaF3G2″GlcT(XP_019424989); PhA3G6″RhaT (CAA81057); PpF3G2″Glc (XP_007213494).

The full-length *Ib3GGT* coding sequence (GenBank accession number EF108571) was identified from a sweet potato cDNA library by comparison with the *At3GGT* sequence. The 1380-bp *Ib3GGT* gene harbors an ORF encoding 459 amino acids (aa) with a calculated molecular mass of 50.87 kDa and an isoelectric point of 6.537. Further sequence analysis of Ib3GGT showed that its amino acid sequence shared the common domain of a PSPG (putative secondary product glycosyltransferase) box (334–377 aa, Fig. 1A) in the C-terminal region with other UF3GGTs that constitute the sugar-donor binding pockets (Osmani *et al.*, 2009). In addition, the sugar-donor specificity has been reported to be partially determined by the last amino acid residue of the PSPG box, namely glutamine (Gln) for UDP-glucose and histidine (His) for UDP-galactose (Kubo *et al.*, 2004). However, the last amino acid residue of the PSPG boxes were Gln in both Ib3GGT and At3GGT but for UDP-glucose and UDP-xylose as the sugar-donor, respectively. This indicated that other amino acid residues in the sequences might contribute towards sugar-donor specificity, and this required further examination.

The phylogenetic analysis showed that Ib3GGT belonged to a cluster of typical further glycosyltransferases, and was most closely related to Ip3GGT of *Ipomoea purpurea* (Morita *et al.*, 2005), showing 94.3% identity (Fig. 1B). Ib3GGT also showed a 45.7% and 45.6% identity to At3GGT and At3GGTF, respectively.

### Ib3GGT catalyses the glycosylation of anthocyanidin 3-*O*-glucoside into anthocyanidin 3-*O*-sophoroside and is highly specific to UDP-glucose

**Fig. 2.**
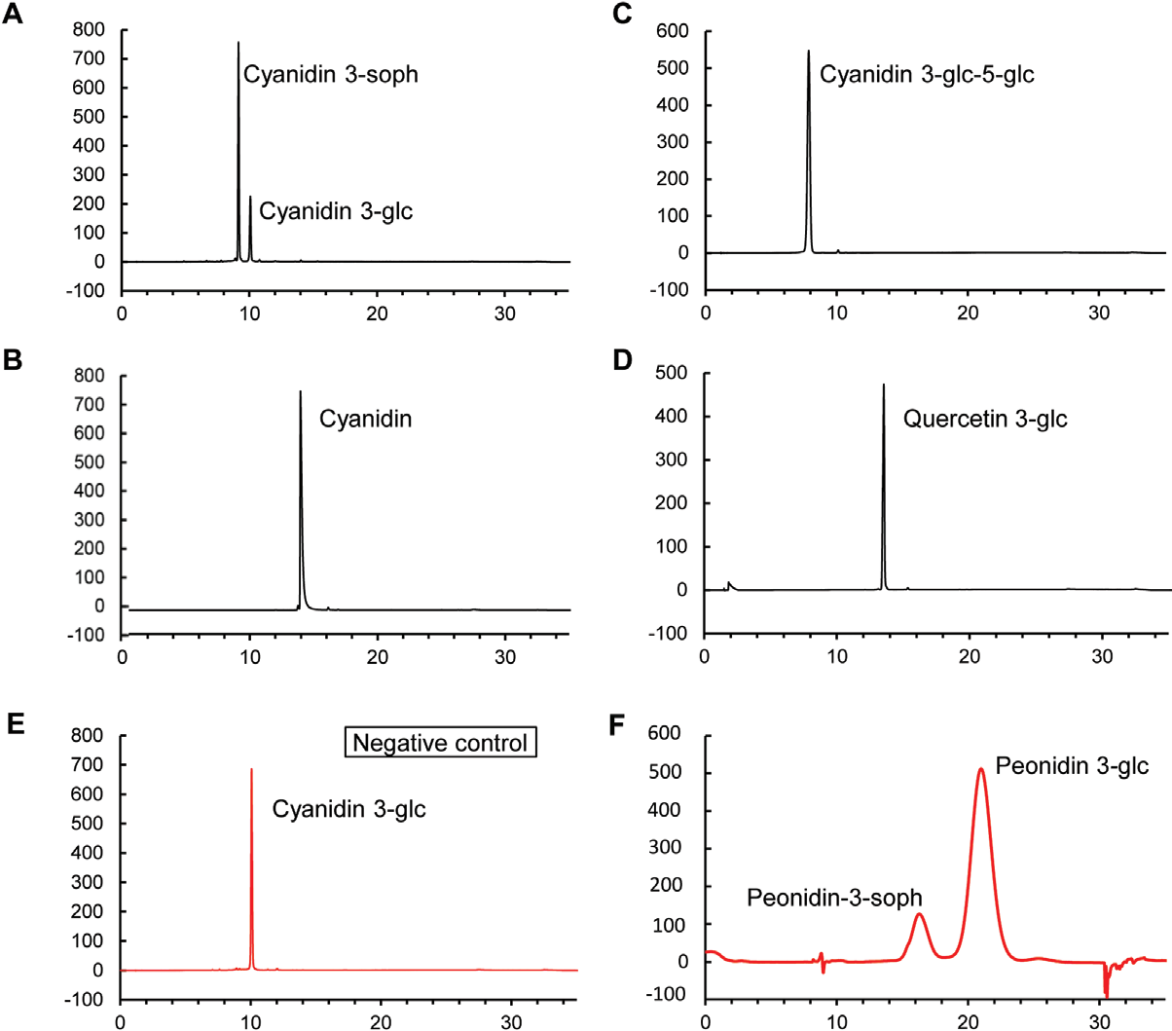
Functional assays of Ib3GGT recombinant protein using UDP-glucose and different acceptor substrates, as determined by HPLC. (A) Cyanidin 3-*O*-glucoside as acceptor substrate and cyanidin 3-*O*-sophoroside as the product; (B) Cyanidin as acceptor substrate; (C) Cyanidin 3,5*-O*-diglucoside as acceptor substrate; and (D) Quercetin 3*-O*-glucoside as acceptor substrate. (E) Cyanidin 3*-O*-glucoside as acceptor substrate without Ib3GGT protein treatment. (F) Peonidin 3*-O*-glucoside as acceptor substrate and peonidin 3-*O*-sophoroside as the product.

To further examine the function of Ib3GGT *in vitro*, recombinant His-tag fusion Ib3GGT proteins in yeast were used to assess the enzymatic activity. The catalysed specificity of Ib3GGT was examined using different sugar acceptors and the donor of UDP-glucose. The recombinant Ib3GGT protein only catalysed the conversion of cyanidin 3*-O*-glucoside into cyanidin 3*-O*-sophoroside (Fig. 2A). In addition, the Ib3GGT protein could use peonidin 3*-O*-glucoside as the glycosyl acceptor to form peonidin 3*-O*-sophoroside (Fig. 2F). Other glucosyl acceptors such as cyanidin, cyanidin 3,5*-O*-diglucoside, and flavonol 3*-O*-glucoside could not serve as substrates, and hence no product was detected (Fig. 2B–D), similar to the negative control (empty vector) (Fig. 2E). These findings indicated that Ib3GGT used anthocyanidin 3*-O*-glucoside as the glycosyl acceptor.

Ib3GGT specificity was also confirmed using UDP-glucose, UDP-xylose, UDP-galactose, and UDP-arabinose as donor substrates (Table 1). The only major UGT activity was with UDP-glucose, indicating that Ib3GGT was highly specific to UDP-glucose, and hence the weak utilization of UDP-xylose indicated a low affinity to this substrate. In contrast, the At3GGT protein was capable of using only UDP-xylose as the sugar donor to catalyse cyanidin 3*-O*-glucoside into cyanidin 3*-O*-glucoside-2″*-O*-xyloside (Table 1; Yonekura-Sakakibara *et al.*, 2012), demonstrating the divergence in the specificity of sugar donors by the two UF3GGTs from different species. Ib3GGT or At3GGT could not catalyse UDP-galactose and UDP-arabinose as sugar donors (Table 1).

**Table 1.** Sugar-substrate specificity of different 3GGT proteins

Sugar donor	Relative activity (%)						
	Ib3GGT	At3GGT	Ib3GGT^T138I^	At3GGT^I142T^	Pp3GGT	La3GGT	Cs3GGT
UDP- glucose	100 ± 8.5	ND	ND	91.0 ± 12.5	100 ± 10.5	100 ± 9.3	ND
UDP- xylose	10.4 ± 1.2	100 ± 15	6.1 ± 0.79	100 ± 18.3	9.4 ± 1.4	11.4 ± 1.2	100 ± 11.7
UDP- galactose	ND	ND	ND	ND	–	–	–
UDP- arabinose	ND	ND	ND	ND	–	–	–

The reactions were performed with cyanidin 3-*O*-glucoside as the sugar acceptor. ND, not detected; –, not tested. The method for calculation of sugar donor specificity was according to Yonekura-Sakakibara *et al.* (2012). Abbreviations for species: At, *Arabidopsis thaliana*; Ib, *Ipomoea batatas*; Pp, *Prunus persica*; La, *Lupinus angustifolius*; Cs, *Camelina sativa*.

### Thr-138 of Ib3GGT contributes to sugar-donor preference

To further identify the key amino acid residue of Ib3GGT responsible for sugar-donor recognition, docking experiments were performed based on the 3D structures of over 10 different glycosyltransferases enzymes from various plants (Shao *et al.*, 2005; Offen *et al.*, 2006; Brazier-Hicks *et al.*, 2007; Modolo *et al.*, 2009; Hiromoto *et al.*, 2013, 2015; Wetterhorn *et al.*, 2016). The overall structures of these glycosyltransferases shared a similar folding topology: two Rossmann-like domains formed a cleft containing two substrate-binding sites with one functional conserved histidine residue located between these sites (Supplementary Fig. S1). By using the Dali server (Holm and Laakso, 2016), more UGT homologous structures were analysed and the root mean square deviations (RMSDs) between them ranged from 1.1–2.5 Å over the core structure region (Supplementary Fig. S2). Because the structures of the sugar donors were similar, the protein structures also shared a group of conserved residues in their binding pockets (Supplementary Fig. S2).

**Fig. 3.**
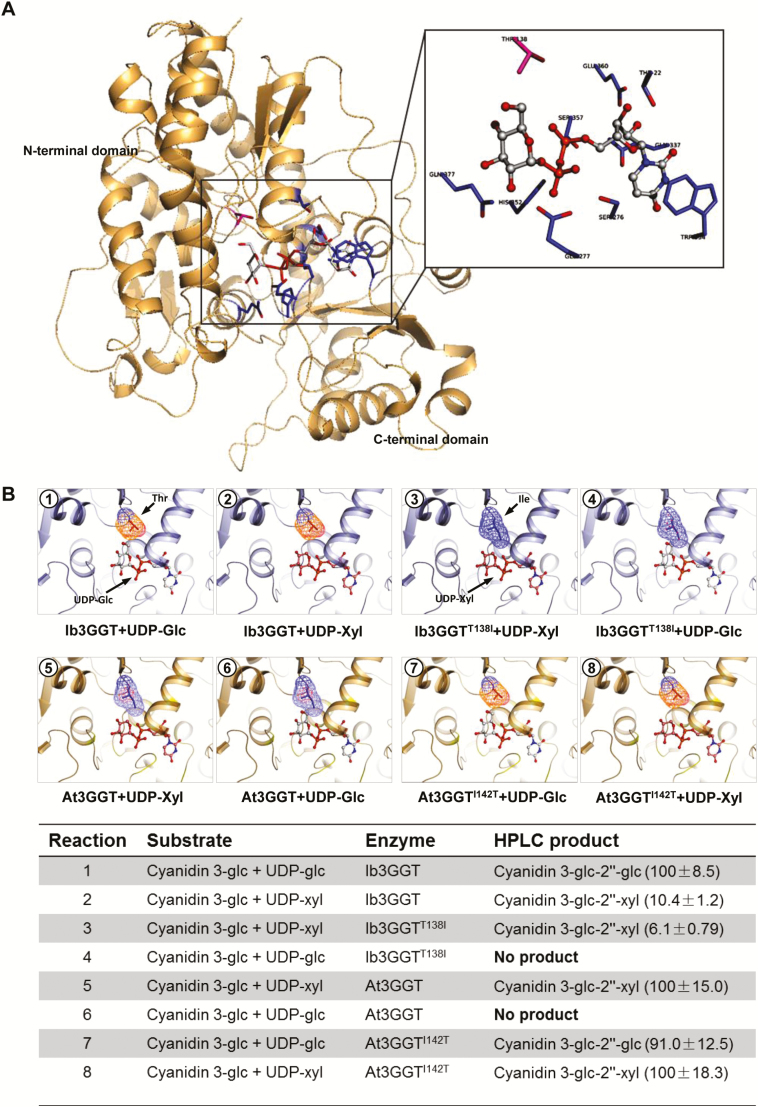
Three-dimensional modeling of Ib3GGT and At3GGT interactions with sugar donors and glycone acceptors. (A) Active center of Ib3GGT showing the key amino acid residues for the sugar donor and acceptor positions. (B) Illustrations of docking of sugar donors and a glycone acceptor in the binding pocket of wild-type and mutant Ib3GGT and At3GGT. The performance of their reactions using cyanidin 3*-O*-glucoside with the sugar nucleotides UDP-glucose or UDP-xylose is shown in the bottom panel. The percentage of relative enzyme activity is indicated in parentheses.

Two different methods, SWISS-MODEL (Biasini *et al.*, 2014) and I-TASSER Suite (Yang *et al.*, 2015), were used for to build models of the structure of Ib3GGT/At3GGT and the results were compared. Both sets of results were similar, with the RMSD between the two Ib3GGT modeled structures being 1.72 Å and that between the two At3GGT modeled structures being 1.92 Å (Supplementary Fig. S3). We selected the SWISS-MODEL service to compare the Ib3GGT/At3GGT structures. In the modeled structure of Ib3GGT, Thr22/Ser276/Glu360/Gln337/Gln338/Trp334 formed a binding pocket and interacted with the uridine group of UDP-glucose (Fig. 3A, Supplementary Fig. S1B); Glu277/His352/Ser357 showed strong interactions with the diphosphate group, (Fig. S1C). Our results indicated that the residues of the binding pocket were conserved, and this is similar to other UGTs (George Thompson *et al.*, 2017; Hsu *et al.*, 2018).

The two modeled structures shared a common group of residues in the sugar-binding pocket (Glu277/Asp376/Gln377 in Ib3GGT and Gln285/Glu385/Gln386 in At3GGT), and the only difference was Thr-138 in Ib3GGT and Ile-142 in At3GGT. In Ib3GGT the distance between *O*-6-glucose and Thr-138 was 2.7 Å, which would allow for a tight interaction between them [for hydrogen bonds, the distance between donor and acceptor atoms is usually 2.6–3.3 Å; hydrophobic interactions (van der Waals bonds) have carbon–carbon distances that are a little larger, usually 3.3–4.0 Å] (Supplementary Fig. S4). However, the distance between the UDP-glucose *O*-6-glucose and Ile 142 in At3GGT was 1.7 Å (Supplementary Fig. S4B), which is too narrow to accept the ligand (the Protein Database Bank defines the ‘magnitude’ of a clash as 2.2 Å). If UDP-glucose were to be replaced with UDP-xylose, this may result in a weak interaction between the xylose group and Thr-138 in Ib3GGT (Supplementary Fig. S4C) and form a 3.9-Å hydrophobic interaction in At3GGT (Supplementary Fig. S4D). These modeling observations were consistent with our enzymatic activity assays (Table 1). Therefore, we hypothesized that the residue Thr-138 in Ib3GGT and its equivalent Ile-142 in At3GGT are the key residues for sugar-donor specificity in purple sweet potato and Arabidopsis, respectively.

To test this hypothesis, we constructed the site-directed mutants Ib3GGT^T138I^ (Thr-138 changed to Ile-138) and At3GGT^I142T^ (Ile-142 changed to Thr-142). Their enzyme activities showed that both the Ib3GGT protein and Ib3GGT^T138I^ could catalyse UDP-xylose to cyanidin 3*-O*-glucoside-2″*-O*-xyloside. However, the Ib3GGT^T138I^ mutant failed to utilize UDP-glucose (Fig. 3B). On the other hand, At3GGT^I142T^ could not only primarily catalyse UDP-xylose but could also use UDP-glucose to synthesize cyanidin 3*-O*-sophorosides. These findings confirmed that Thr-138 is a key residue for sugar (glucose/xylose) recognition in Ib3GGT.

To ascertain whether Thr-138 is a key residue for other sugar recognition, 3D models generated for UDP-galactose and UDP-arabinose were compared in the same position with UDP-glucose and UDP-xylose. UDP-galactose should have less binding affinity than UDP-glucose because of the difference in the direction of the fifth-position oxygen atom, which changes the distance between it and the main-chain N from 3.1 Å to 4.93 Å (Supplementary Fig. S5A, B). UDP-arabinose also has less binding affinity than UDP-xylose because of the disappearance of the interaction between the first-position oxygen atom with His-20 (Supplementary Fig. S5C, D). As expected, no enzymatic activities were detected for Ib3GGT or Ib3GGT ^T138I^ using UDP-galactose and UDP-arabinose as sugar donors (Table 1).

To verify whether other species have the same mechanism of sugar-donor selectivity, two 3GGT proteins containing the Thr-138 residue from *Prunus persica* (Pp3GGT, XP_007213494) and *Lupinus angustifolius* (La3GGT, XP_019424989) and one containing Ile-138 from *Camelina sativa* (Cs3GGT, XP_018450414) were cloned (Supplementary Fig. S6). Both Pp3GGT and La3GGT preferred UDP-glucose rather than UDP-xylose as the sugar donor (Table 1). Their weak utilization of UDP-xylose indicated a low affinity to this substrate. In contrast, the Cs3GGT protein was capable of using only UDP-xylose as the sugar donor (Table 1). Therefore, the Thr-138 residue plays a key role in the specificity for UDP-glucose donors by the two kinds of UF3GGTs. These results indicated that plant UGTs may share a common mechanism in sugar-donor selectivity.

### *Ib3GGT* expression in Arabidopsis produces new anthocyanin molecules

**Fig. 4.**
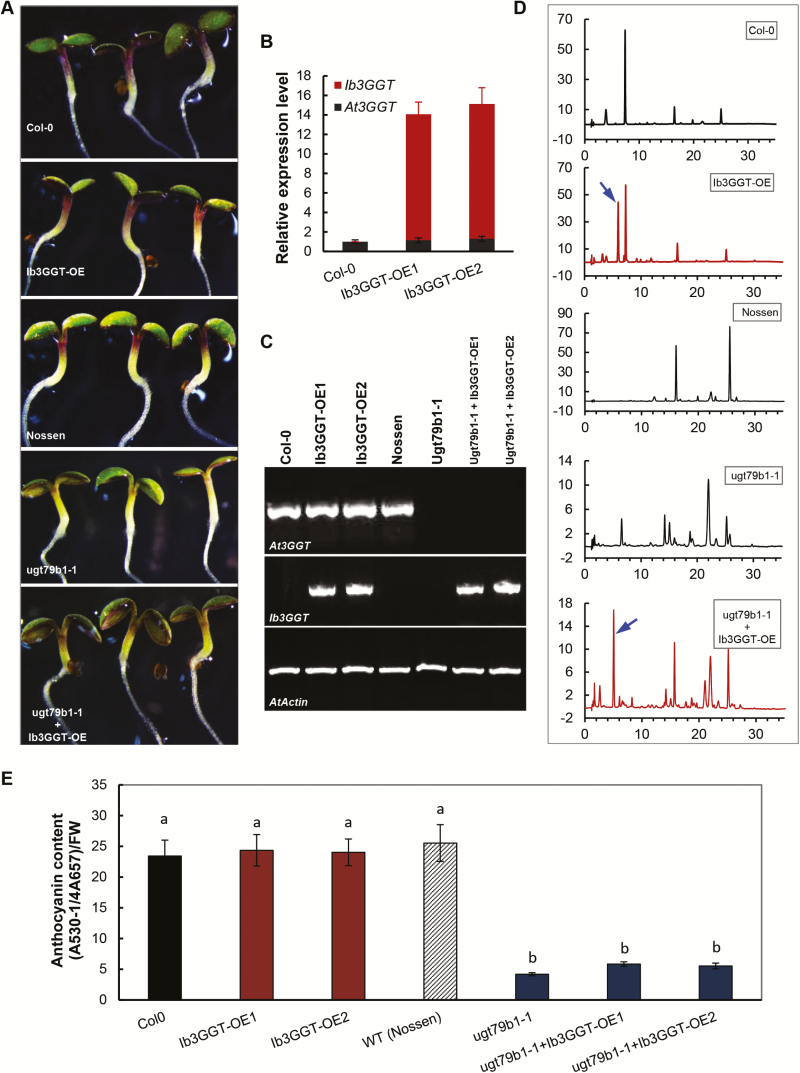
Anthocyanin characterization of transgenic Arabidopsis plants overexpressing *Ib3GGT* gene. (A) Anthocyanin pigmentation in seedlings of wild-types (WT, Col-0 and Nossen), *Ib3GGT*-overexpressing Col-0 lines (Ib3GGT-OE), the *ugt79b1* mutant (ugt79b1-1), and Ib3GGT-overexpressing *ugt79b1* line (ugt79b1-1+Ib3GGT-OE). (B) The expression of *At3GGT* and *Ib3GGT* in the WT Col-0 and *Ib3GGT*-overexpressing Col-0 lines (Ib3GGT-OE1 and Ib3GGT-OE2) as determined by real-time RT-PCR analysis. *Actin* was used as the reference gene. (C) RT-PCR detection of *At3GGT* and *Ib3GGT* expression in the WTs (Col-0 and Nossen), two independent *Ib3GGT*-overexpressing Col-0 lines (Ib3GGT-OE1 and Ib3GGT-OE2), the *ugt79b1* mutant, and two *Ib3GGT*-overexpressing *ugt79b1* lines (ugt79b1-1+Ib3GGT-OE1 and ugt79b1-1+Ib3GGT-OE2). (D) Anthocyanin component profiles as determined by HPLC/PDA/MS in the seedlings of WTs (Col-0 and Nossen), the *ugt79b1* mutant, and Ib3GGT-overexpressing *ugt79b1* line (ugt79b1-1+Ib3GGT-OE). The arrows indicate the new peaks of cyanidin 3*-O*-sophoroside. (E) Anthocyanin content in the WTs (Col-0 and Nossen), two independent *Ib3GGT*-overexpressing Col-0 lines (Ib3GGT-OE1 and Ib3GGT-OE2), the *ugt79b1* mutant, and two *Ib3GGT*-overexpressing *ugt79b1* line (ugt79b1-1+Ib3GGT-OE1 and ugt79b1-1+Ib3GGT-OE2). Different letters indicate significant differences (one-way ANOVA, *P*<0.05).

To further validate the activity of Ib3GGT *in planta*, the *Ib3GGT* gene was overexpressed in Arabidopsis Col-0 and in the UGT79B1 transposon insertion mutants *ugt79b1-1* and *ugt79b1-2* (Kuromori *et al.*, 2004; Ito *et al.*, 2005). RT-PCR analysis confirmed the overexpression of *Ib3GGT* in these plants (Fig. 4B, C). When compared to the WT, the transgenic lines (T3 homozygous Ib3GGT-OE and *Ib3GGT* overexpression of *ugt79b1-1* and *ugt79b1-2*) showed a new peak with an *m/z* value corresponding to cyanidin 3*-O*-sophoroside, as detected by HPLC-electrospray ionization (ESI)-tandem MS analysis (Fig. 4D, Supplementary Fig. S7). This showed that both the transgenic lines produced cyanidin 3*-O*-sophoroside, although the purple-color phenotype and anthocyanin content at the cotyledon-stage seedling in Ib3GGT-OE was indistinguishable from the WT (Fig. 4A, E). The seedlings of the *ugt79b1-1* and ugt79b1-2 lines, which lacked the purple coloration as compared to the WT Nossen, showed partly recovered anthocyanin accumulation when overexpressing the *Ib3GGT* gene (Fig. 4A, bottom panel, Supplementary Fig. S7). These results confirmed that Ib3GGT could specifically catalyse the conversion of cyanidin 3*-O*-glucoside to cyanidin 3*-O*-sophoroside in Arabidopsis, a biological process normally absent in this species.

### *Ib3GGT* expression is associated with anthocyanin accumulation and organ development, and is regulated by IbMYB1

**Fig. 5.**
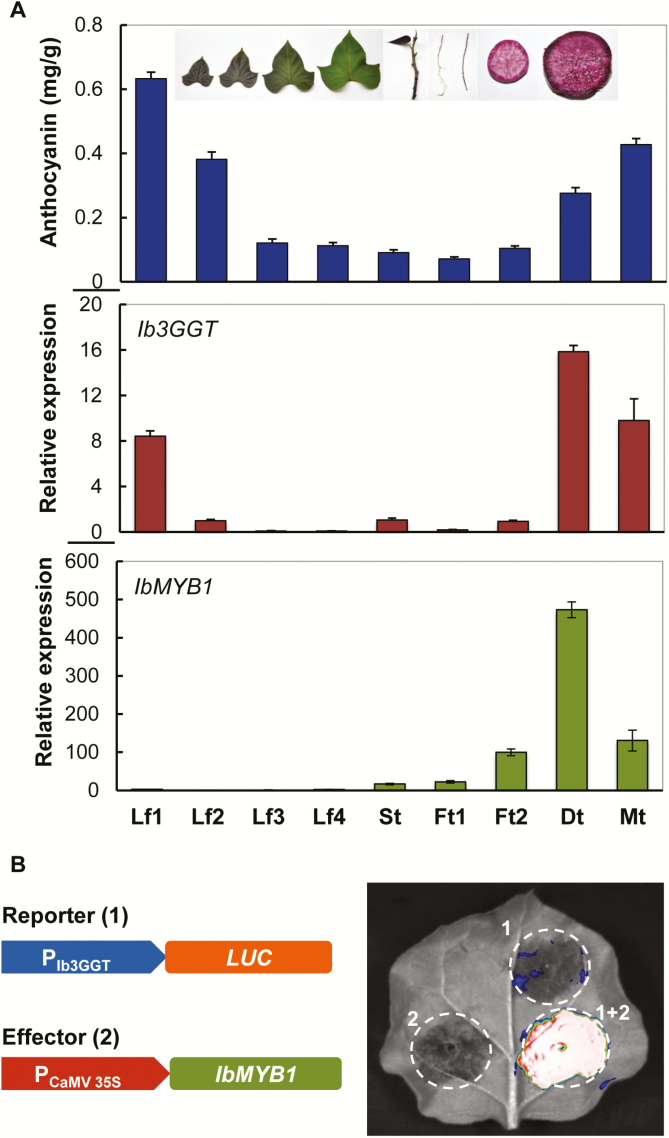
Correlation of anthocyanin accumulation and gene expression in various organs of sweet potato plants. (A) Profiles of anthocyanin accumulation, and *Ib3GGT* and *IbMYB1* transcript levels as detected by qRT-PCR. Lf1, Lf2, Lf3, and Lf4 represent leaves of different developmental stages (as illustrated); St, stem; Ft1, white fibrous root; Ft2, red fibrous root; Dt, developing root; Mt, mature root. Values are means (±SD) (*n*=6). (B) Luciferase (LUC) assay of *Ib3GGT* promoter activity (reporter) regulated by IbMBY1 (effector) in agro-infiltrated *Nicotiana benthamiana* leaves.

Anthocyanin accumulation in sweet potato plants showed an organ-dependent pattern. Immature leaves and mature storage roots contained the highest levels of anthocyanins, while mature leaves and fibrous roots had the lowest (Fig. 5A). Among the leaves, the least mature (Lf1) had an anthocyanin concentration of 0.6324 mg g^–1^, ~7-fold higher than that of the most mature (Lf4). *Ib3GGT* expression analysed by real-time PCR also showed a similar pattern in the different organs, with high expression in immature leaves as well as in developing and mature storage roots. *Ib3GGT* was more highly expressed in developing storage roots (Fig. 5A, Dt) as compared to mature roots (Mt), although the latter accumulated 30% more anthocyanins (0.4276 mg g^–1^). Overall, *Ib3GGT* expression was associated with anthocyanin accumulation in the different organs of the plants.

The transcription factor IbMYB1 is the main regulator of anthocyanin biosynthesis in tuberous roots of purple sweet potato (Mano *et al.*, 2007), and we found that its expression in the roots was associated with anthocyanin accumulation (Fig. 5A). Therefore, we hypothesized that IbMYB1 regulates the expression of *Ib3GGT* by binding to the G-box element (CACGTG) in the promoter (Mano *et al.*, 2007). The promoter region of *Ib3GGT* showed a G-box element at position 992, as analysed by the PlantCARE software (Supplementary Fig. S8). To confirm that *Ib3GGT* expression was regulated by IbMYB1, a LUC gene reporter driven by a 2000-bp promoter of *Ib3GGT* was assayed for activity in tobacco leaves after co-agro-infiltration with the effector, which harbored the *CaMV 35S::IbMYB1* expression cassette. Paired vectors containing the *Ib3GGT* promoter and *CaMV 35S::IbMYB1* resulted in strong luciferase activity whereas the corresponding empty vectors failed to detect luminescent signals (Fig. 5B). These findings indicated that IbMYB1 regulates the expression of *Ib3GGT* in sweet potato plants.

### Regulation of *Ib3GGT* expression in sweet potato alters the total anthocyanin content but not the overall component profile

**Fig. 6.**
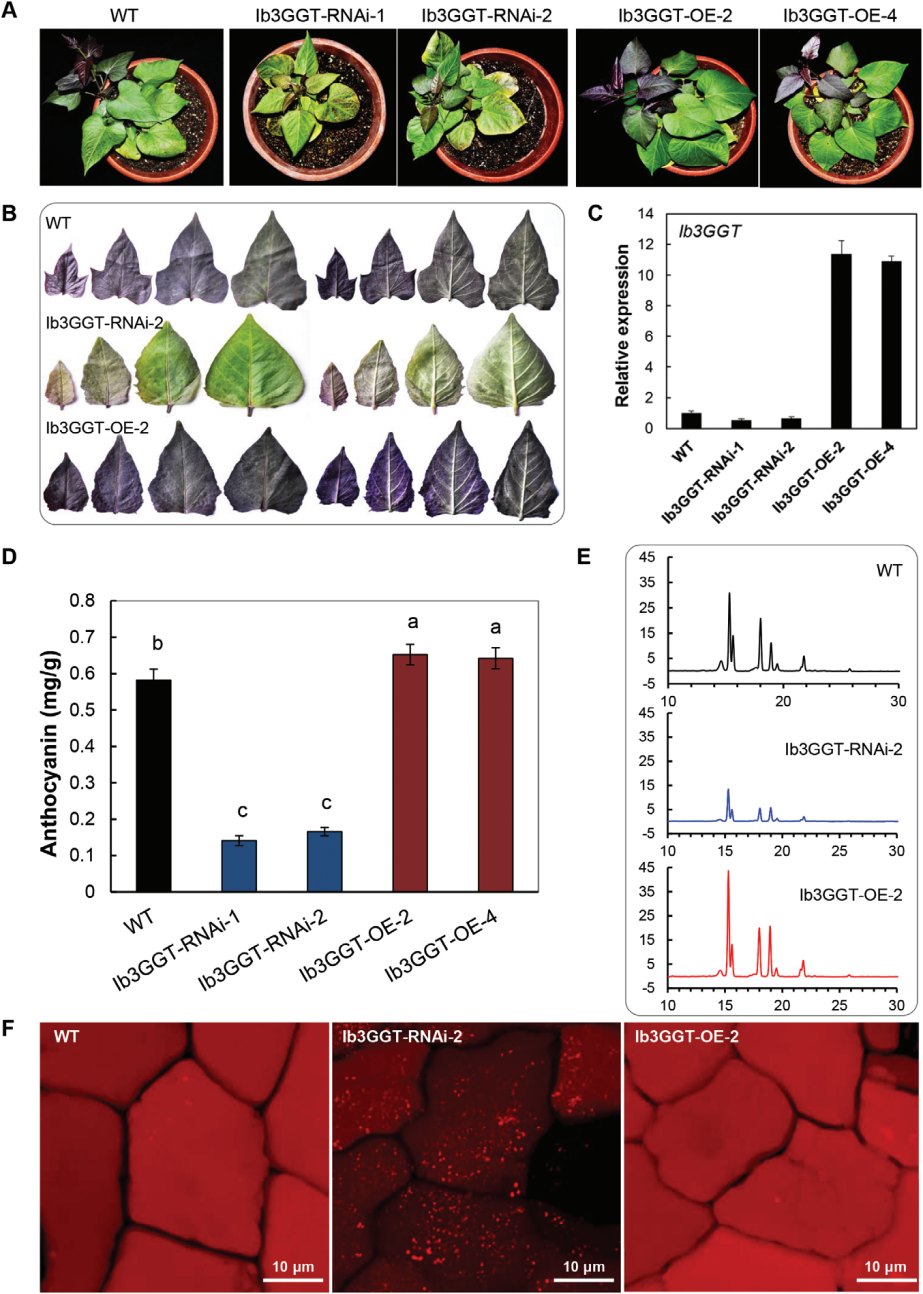
Anthocyanin characterization of wild-type and *Ib3GGT* transgenic sweet potato plants. (A) Plant phenotypes. WT, wild type; Ib3GGT-OE line, transgenic plants overexpressing *Ib3GGT*; Ib3GGT-RNAi line, *Ib3GGT* RNAi transgenic plants. (B) Anthocyanin pigmentation in the top leaves of WT, Ib3GGT-RNAi-2, and Ib3GGT-OE-2 plants. Both the adaxial (left) and abaxial (right) leaf surfaces are shown. (C) Relative transcription levels of native *Ib3GGT* and the *Ib3GGT* transgene in WT and transgenic lines assessed as by qRT-PCR. *Actin* was used as the reference gene. (D) Anthocyanin content in WT, Ib3GGT-RNAi, and Ib3GGT-OE plant lines. Different letters indicate significant differences (one-way ANOVA, *P*<0.05). (E) Component profiles of anthocyanins in WT, Ib3GGT-RNAi-2, and Ib3GGT-OE-2 plants, as assessed by HPLC. (F) Anthocyanin autofluorescence in leaf epidermal cells of Ib3GGT-RNAi-2 and Ib3GGT-OE-2 plants.

To further elucidate the role of Ib3GGT in sweet potato, *Ib3GGT*-overexpressing (Ib3GGT-OE) or -RNAi (Ib3GGT-RNAi) transgenic plants were analysed. The expression of *Ib3GGT* was decreased in the Ib3GGT-RNAi lines and increased in the Ib3GGT-OE lines, as confirmed by the phenotype (Fig. 6A, B) and real-time PCR analyses (Fig. 6C). Compared to the WT, the Ib3GGT-RNAi lines showed reduced anthocyanin levels in the leaves, whereas Ib3GGT-OE plants showed an increased anthocyanin accumulation in the top leaves (Fig. 6A, B). The level of anthocyanin in the third leaf was reduced to 28.5% of the WT in Ib3GGT-RNAi-2 and increased to 112% in Ib3GGT-OE-2 (Fig. 6A, B, D). The changes in the anthocyanin levels were correlated with *Ib3GGT* expression in these plants (Fig. 6C, D). In addition, auto-fluorescence assays in leaf epidermal cells showed a dramatic reduction in the fluorescent intensity in the Ib3GGT-RNAi lines (dull intensity), further indicating lower anthocyanin levels, while the WT and Ib3GGT-OE transgenic plants displayed strong signals (bright intensity) (Fig. 6F). Nevertheless, the overall profile of the anthocyanins was not altered in these plants (Fig. 6E), indicating that Ib3GGT is involved in an early stage of anthocyanin modification. Similar trends of altered anthocyanin accumulation were also observed in corresponding field-grown plants (Supplementary Fig. S9).

### Localization of Ib3GGT in the cytosol

**Fig. 7.**
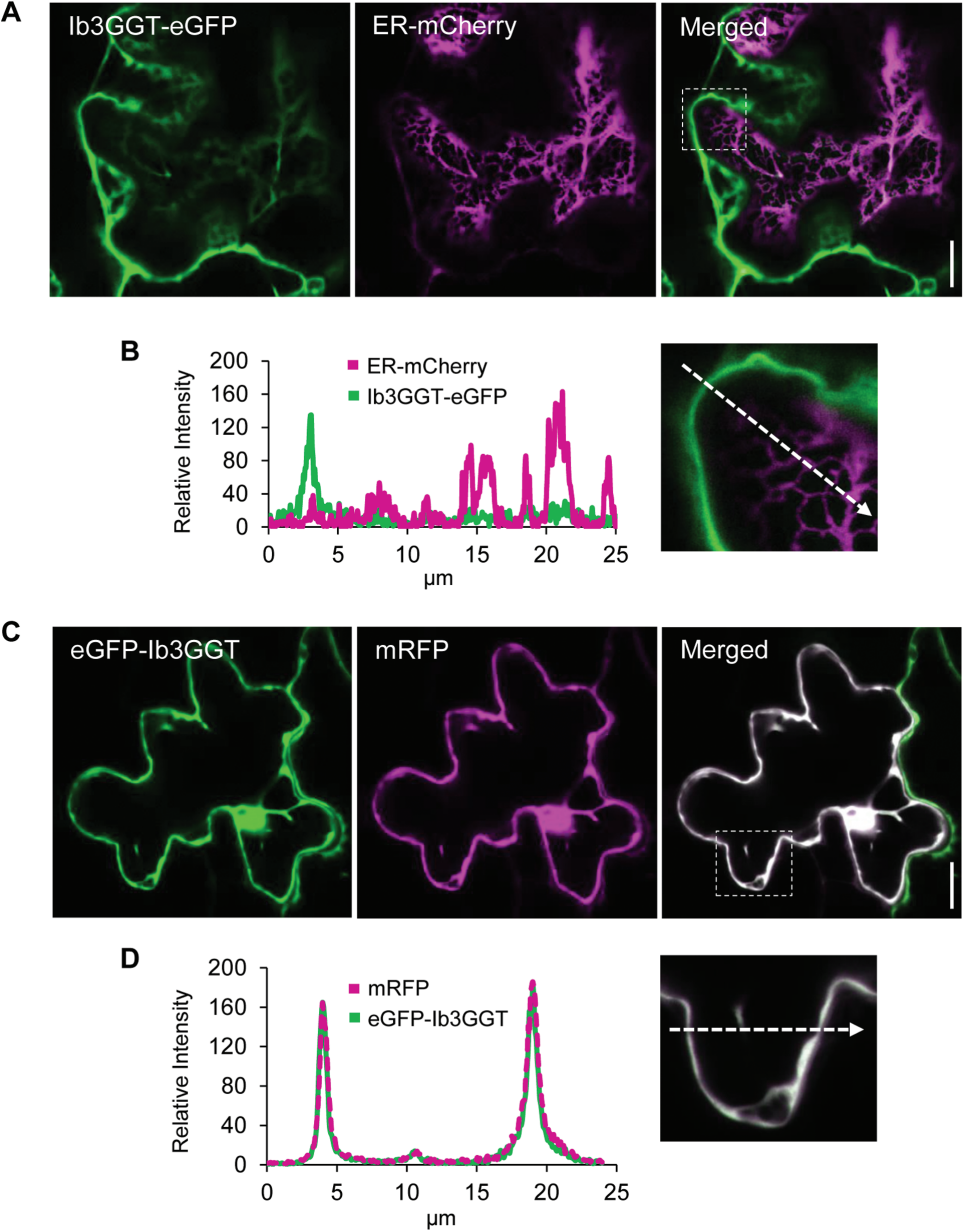
Subcellular localization of Ib3GGT after transient expression in *Nicotiana benthamiana* leaves. (A) Optical sections through a pavement cell, co-expressing Ib3GGT-eGFP and ER-mCherry. The GFP and mCherry signals are distinct in the merged image. (B) Magnified image of the region outlined in the merged panel in (A) and the relative fluorescence intensity along the axis marked by the dashed arrow. (C) Co-expression of eGFP-Ib3GGT and soluble mRFP. The white pixels of the merged image show an overlay of both channels. (D) Magnified image of the region outlined in the merged panel in (C) and the relative fluorescence intensity along the axis marked by the dashed arrow. eGFP-Ib3GGT and soluble mRFP are co-localized in the cytoplasm. Scale bars are 15 µm.

We were unable to predict the localization of the Ib3GGT protein because no signal peptides were found in the full protein sequence using the SignalP 3.0 Server (http://www.cbs.dtu.dk/services/SignalP-3.0/). Anthocyanins have been suggested to be synthesized on the outer surface of the ER (Poustka *et al.*, 2007). To examine the localization of Ib3GGT, the N- and C-terminals of Ib3GGT fused with eGFP, with an ER-marker, or with a soluble mRFP were transiently expressed in leaves of *N. benthamiana* (Fig. 7). Both the fluorescent signals of the N- and C-terminal Ib3GGT fusion GFP proteins were found to localize in the cytosol, similar to the soluble mRFP (Fig. 7C, D). In addition, when Ib3GGT-eGFP was expressed together with ER-mCherry, no co-localization was found (Fig. 7A, B). Thus, Ib3GGT is a soluble protein in the cytosol and not associated with the ER. However, some signal of the eGFP-Ib3GGT fusions was also observed in the nucleus.

## Discussion

### Glycosyl extension modifications of anthocyanins in the cytosol

Glycosyl extension of anthocyanins is an essential step in their biosynthesis, accumulation, and stability (Yonekura-Sakakibara *et al.*, 2008; Zhang *et al.*, 2014). In purple sweet potato the major anthocyanins include cyanidin and peonidin 3-sophorosides as well as their acylated derivatives (Truong *et al.*, 2010; Lee *et al.*, 2013), indicating that glycosyl extension is required for the conversion from anthocyanidin 3*-O*-glucosides into anthocyanidin 3*-O*-sophorosides. In this study, we found that Ib3GGT was responsible for this reaction using UDP-glucose as the sugar donor. Unlike sweet potato, in Arabidopsis glycosyl extension of anthocyanins is performed by xylosylation catalysed by UGT79B1 (Saito *et al.*, 2013). Our results showed that Arabidopsis lacks the enzymes to form cyanidin 3*-O*-sophoroside using cyanidin 3*-O*-glucosides as the potential substrate for further modification, as overexpressing Ib3GGT in Arabidopsis only showed a peak of cyanidin 3*-O*-sophoroside without a change in other anthocyanin components. In sweet potato, Ib3GGT is of key importance in anthocyanin metabolism because of its role in glycosyl extension modifications.

Glycosyl extension is a critical step in determining subsequent anthocyanin modifications, such as malonylation and acylation (Yonekura-Sakakibara *et al.*, 2008; Andersen and Jordheim, 2010). As a UDP-glycosyltransferase, Ib3GGT can add a sugar residue to anthocyanidin 3-*O*-glucosides but not to anthocyanidin 3,5-*O*-diglucosides (Fig. 2A, C). Thus, we conclude that a UDP-glucose-dependent anthocyanin 5-*O*-glucosyltransferase (UA5GT) catalyses a glucose molecule into the 5th position of the C-ring that can hinder the transfer of a glucose molecule into the 2″ position of anthocyanidin 3-*O*-glucosides. This phenomenon may be a critical factor in determining subsequent steps in the modification of anthocyanins. Ib3GGT cannot catalyse flavonol-3-*O*-glucoside as an acceptor substrate, demonstrating that Ib3GGT has substrate-specificity in purple sweet potato (Fig. 2D). However, At3GGT can catalyse flavonol-3-*O*-glucoside and anthocyanin-3-*O*-glucoside as less-specific substrates in Arabidopsis (Saito *et al.*, 2013).

As a primary mechanism that maintains metabolic homeostasis in plants, glycosylation contributes to the diversity of synthesis of various secondary metabolites, thereby altering the biological functions of these metabolites (Jones and Vogt, 2001; Gachon *et al.*, 2005). Divergence has occurred among species by adaption of glycosyltransferase substrate-specificity. In peach, PpUGT79B performs glycosylation by adding a rhamnoside molecule to anthocyanidin 3-*O*-glucosides to form the anthocyanidin 3-*O*-rutinoside (Cheng *et al.*, 2014). Ib3GGT was functional in transgenic Arabidopsis and produced anthocyanin-3-sophoroside, while the overexpression of UGT79B1 (At3GGT) in sweet potato did not catalyse the production of anthocyanin 3*-O*-glucoside-2″*-O*-xylose (Supplementary Fig. S10). These findings indicated that anthocyanin glycosylation in sweet potato has diverged from that of Arabidopsis towards a specific sugar acceptor. Interestingly, the glycosyl extension of anthocyanin occurs in the cytosol, unlike other UGTs that are mainly ER membrane-bound enzymes (Poustka *et al.*, 2007; Zhao, 2015).

### Key amino acids in UGTs affect both sugar-donor preference and regioselectivity

The phylogenetic comparison of flavonoid GGTs suggested that potentially conserved amino acid residues are involved in further substrate-selectivity (Fig. 1). Four amino acid residues (Trp-334/Gln-337/Glu-360/His-352 in Ib3GGT) were generally conserved across all known flavonoid 3*-O*-glycoside-2″*-O*-glycosyltransferases. The close relationship between Ib3GGT and UGT79B1 in the phylogenetic tree also indicated that the sugar-donor selectivity of flavonoid GGTs was established after species differentiation (Saito *et al.*, 2013). In sweet potato, Ib3GGT accepts UDP-glucose as the sugar donor to conjugate to anthocyanins such as cyanidin 3-*O*-glucoside or peonidin 3-*O*-glucoside. Interestingly, Arabidopsis also has a UDP-glucose:flavonoid 3*-O*-glucoside-2″*-O*-glucosyltransferase (At3GGTF), which preferentially uses flavonol 3*-O*-glucoside and UDP-glucose as substrates (Kubo *et al.*, 2007). Thr-138, as the key residue for UDP-glucose recognition, was also conserved in glucosyltransferases that use UDP-glucose as the sugar donor in morning glory, Arabidopsis (At3GGTF), *Ricinus communis*, and *Glycine max* (Supplementary Fig. S6). Corresponding to the Thr-138 residue, Ile-142 was the residue for UDP-xylose recognition in Arabidopsis. The corresponding sites of Ile in *Camelina sativa*, Thr in *Tarenaya hassleriana* and *Brassica napus*, and Val in *Eucalyptus grandis* were also responsible for recognizing UDP-xylose (Supplementary Fig. S6).

### Anthocyanin glycosyltransferases are regulated by transcription factors

Anthocyanin biosynthesis is a finely regulated system involving multiple transcription factors (TFs) associated with plant development (Pireyre and Burow, 2015; Xu *et al.*, 2015). For example, the temporal and spatial regulation of anthocyanin production in flowers is mediated by the TFs R2R3-MYB, basic Helix-Loop-Helix (bHLH), or WD40 types (reviewed in Davies *et al.*, 2012). To date, the only well-characterized TF in sweet potato is R2R3-MYB type IbMYB1, which controls anthocyanin biosynthesis specifically in tuberous roots by inducing structural anthocyanin genes (Mano *et al.*, 2007). In our study, the accumulation of anthocyanin in different organs was strongly associated with the expression of *Ib3GGT* (Fig. 5), which indicated its divergent modifications during plant development. Importantly, the activation of the promoter by IbMYB1 confirmed that *Ib3GGT* was highly regulated by this TF in storage roots. The relatively low levels of *IbMYB1* transcripts in the leaves might reflect its tissue specificity. In Arabidopsis, it is well-documented that the R2R3-MYB TF can induce glycosyltransferases such as UGT79B1 (Tohge *et al.*, 2005; Yonekura-Sakakibara *et al.*, 2008; Stracke *et al.*, 2010).

In summary, sweet potato Ib3GGT catalyses anthocyanidin 3-*O*-glucosides into anthocyanidin 3-*O*-sophorosides using UDP-glucose as a sugar donor. The Thr-138 of Ib3GGT is a key residue for sugar-donor selectivity in the glycosyl extension that contributes to the stability and diversity of anthocyanins. Ib3GGT glycosylation occurs in the cytosol and is regulated by the IbMYB1 TF. This study thus provides insights regarding the glycosylation enzymes involved in the divergence of secondary metabolism that can assist in developing a useful approach towards diversifying certain flavonoids in crop plants.

## Supplementary data

Supplementary data are available at *JXB* online.

Fig. S1. UDP-glucose binding sites of Ib3GGT.

Fig. S2. Structure alignment of UGT homologs.

Fig. S3. Sequence alignment of Ib3GGT with 2VG8 and 2VCH proteins using SWISS-MODEL and I-TASSER.

Fig. S4. Sugar-donor binding sites in Ib3GGT and At3GGT.

Fig. S5. Difference in binding affinity of sugar analogs.

Fig. S6. Amino acid sequence comparison of GGT analogs with Ib3GGT.

Fig. S7. Anthocyanin pigmentation and component profiles in seedlings of the *ugt79b1-2* mutant and *Ib3GGT*-overexpressing *ugt79b1-2* transgenic line.

Fig. S8. *Ib3GGT* promoter sequence showing the two G-box sites.

Fig. S9. Leaf and root phenotypes of field-grown wild-type, Ib3GGT-RNAi-2 and Ib3GGT-OE-2 plant lines.

Fig. S10. Analysis of anthocyanin compounds in wild-type and *At3GGT*-overexpressing sweet potato plants as determined by HPLC-MS.

Table S1. Anthocyanin compounds in sweet potato and Arabidopsis.

Table S2. List of primers for gene expression analysis in *Arabidopsis* and sweet potato plant lines.

Supplementary Figures S1-S10 and Tables S1-S2

## References

[CIT0001] Andersen ØM, Jordheim M 2010 Anthocyanins. In: Encyclopedia of Life Sciences. Chichester: John Wiley and Sons Ltd. doi:10.1002/9780470015902.a0001909.pub2.

[CIT0002] Biasini M, Bienert S, Waterhouse A, et al 2014 SWISS-MODEL: modelling protein tertiary and quaternary structure using evolutionary information. Nucleic Acids Research 42, W252–W258. 24782522 10.1093/nar/gku340 PMC4086089

[CIT0003] Brazier-Hicks M, Offen WA, Gershater MC, Revett TJ, Lim EK, Bowles DJ, Davies GJ, Edwards R 2007 Characterization and engineering of the bifunctional N- and O-glucosyltransferase involved in xenobiotic metabolism in plants. Proceedings of the National Academy of Sciences, USA 104, 20238–20243. 10.1073/pnas.0706421104 PMC2154415 18077347

[CIT0004] Breton C, Snajdrová L, Jeanneau C, Koca J, Imberty A 2006 Structures and mechanisms of glycosyltransferases. Glycobiology 16, 29R–37R. 16037492 10.1093/glycob/cwj016

[CIT0005] Caputi L, Malnoy M, Goremykin V, Nikiforova S, Martens S 2012 A genome-wide phylogenetic reconstruction of family 1 UDP-glycosyltransferases revealed the expansion of the family during the adaptation of plants to life on land. The Plant Journal 69, 1030–1042. 22077743 10.1111/j.1365-313X.2011.04853.x

[CIT0006] Cheng J, Wei G, Zhou H, Gu C, Vimolmangkang S, Liao L, Han Y 2014 Unraveling the mechanism underlying the glycosylation and methylation of anthocyanins in peach. Plant Physiology 166, 1044–1058. 25106821 10.1104/pp.114.246876 PMC4213075

[CIT0007] Claudia C, Philippe R, Edith F, Vincent B, Yumei Z, Sharyn EP, Juan JR, Martin Y, Christophe D 2017 The class III peroxidase PRX17 is a direct target of the MADS-box transcription factor AGAMOUS-LIKE15 (AGL15) and participates in lignified tissue formation. New Phytologist 213, 250–263. 27513887 10.1111/nph.14127

[CIT0008] Clough SJ, Bent AF 1998 Floral dip: a simplified method for *Agrobacterium*-mediated transformation of *Arabidopsis thaliana*. The Plant Journal 16, 735–743. 10069079 10.1046/j.1365-313x.1998.00343.x

[CIT0009] Davies KM, Albert NW, Schwinn KE 2012 From landing lights to mimicry: the molecular regulation of flower colouration and mechanisms for pigmentation patterning. Functional Plant Biology 39, 619–638. 10.1071/FP12195 32480814

[CIT0010] de Pascual-Teresa S, Sanchez-Ballesta MT 2008 Anthocyanins: from plant to health. Phytochemistry Reviews 7, 281–299.

[CIT0011] Emsley P, Lohkamp B, Scott WG, Cowtan K 2010 Features and development of Coot. Acta Crystallographica. Section D, Biological Crystallography 66, 486–501. 20383002 10.1107/S0907444910007493 PMC2852313

[CIT0012] Fedoroff NV, Furtek DB, Nelson OE 1984 Cloning of the bronze locus in maize by a simple and generalizable procedure using the transposable controlling element Activator (Ac). Proceedings of the National Academy of Sciences, USA 81, 3825–3829. 10.1073/pnas.81.12.3825 PMC345313 16593478

[CIT0013] Gachon CM, Langlois-Meurinne M, Saindrenan P 2005 Plant secondary metabolism glycosyltransferases: the emerging functional analysis. Trends in Plant Science 10, 542–549. 16214386 10.1016/j.tplants.2005.09.007

[CIT0014] George Thompson AM, Iancu CV, Neet KE, Dean JV, Choe JY 2017 Differences in salicylic acid glucose conjugations by UGT74F1 and UGT74F2 from *Arabidopsis thaliana*. Scientific Reports 7, 46629. 28425481 10.1038/srep46629 PMC5397973

[CIT0015] Glover BJ, Martin C 2012 Anthocyanins. Current Biology 22, R147–R150. 22401890 10.1016/j.cub.2012.01.021

[CIT0016] Griesser M, Hoffmann T, Bellido ML, Rosati C, Fink B, Kurtzer R, Aharoni A, Muñoz-Blanco J, Schwab W 2008a Redirection of flavonoid biosynthesis through the down-regulation of an anthocyanidin glucosyltransferase in ripening strawberry fruit. Plant Physiology 146, 1528–1539. 18258692 10.1104/pp.107.114280 PMC2287331

[CIT0017] Griesser M, Vitzthum F, Fink B, Bellido ML, Raasch C, Munoz-Blanco J, Schwab W 2008b Multi-substrate flavonol O-glucosyltransferases from strawberry (*Fragaria* × *ananassa*) achene and receptacle. Journal of Experimental Botany 59, 2611–2625. 18487633 10.1093/jxb/ern117 PMC2486459

[CIT0018] He J, Giusti MM 2010 Anthocyanins: natural colorants with health-promoting properties. Annual Review of Food Science and Technology 1, 163–187. 10.1146/annurev.food.080708.100754 22129334

[CIT0019] He ZH, Cheeseman I, He D, Kohorn BD 1999 A cluster of five cell wall-associated receptor kinase genes, *Wak1–5*, are expressed in specific organs of Arabidopsis. Plant Molecular Biology 39, 1189–1196. 10380805 10.1023/a:1006197318246

[CIT0020] Hiromoto T, Honjo E, Noda N, Tamada T, Kazuma K, Suzuki M, Blaber M, Kuroki R 2015 Structural basis for acceptor-substrate recognition of UDP-glucose: anthocyanidin 3-O-glucosyltransferase from *Clitoria ternatea*. Protein Science 24, 395–407. 25556637 10.1002/pro.2630 PMC4353365

[CIT0021] Hiromoto T, Honjo E, Tamada T, Noda N, Kazuma K, Suzuki M, Kuroki R 2013 Crystal structure of UDP-glucose:anthocyanidin 3-O-glucosyltransferase from *Clitoria ternatea*. Journal of Synchrotron Radiation 20, 894–898. 24121335 10.1107/S0909049513020712 PMC3795551

[CIT0022] Holm L, Laakso LM 2016 Dali server update. Nucleic Acids Research 44, W351–W355. 27131377 10.1093/nar/gkw357 PMC4987910

[CIT0023] Hsu TM, Welner DH, Russ ZN, Cervantes B, Prathuri RL, Adams PD, Dueber JE 2018 Employing a biochemical protecting group for a sustainable indigo dyeing strategy. Nature Chemical Biology 14, 256–261. 29309053 10.1038/nchembio.2552 PMC5866135

[CIT0024] Ito T, Motohashi R, Kuromori T, Noutoshi Y, Seki M, Kamiya A, Mizukado S, Sakurai T, Shinozaki K 2005 A resource of 5814 dissociation transposon-tagged and sequence-indexed lines of Arabidopsis transposed from start loci on chromosome 5. Plant & Cell Physiology 46, 1149–1153. 15840642 10.1093/pcp/pci112

[CIT0025] Jones P, Vogt T 2001 Glycosyltransferases in secondary plant metabolism: tranquilizers and stimulant controllers. Planta 213, 164–174. 11469580 10.1007/s004250000492

[CIT0026] Kim HS, Kim BG, Sung S, Kim M, Mok H, Chong Y, Ahn JH 2013 Engineering flavonoid glycosyltransferases for enhanced catalytic efficiency and extended sugar-donor selectivity. Planta 238, 683–693. 23801300 10.1007/s00425-013-1922-0

[CIT0027] Kovinich N, Kayanja G, Chanoca A, Riedl K, Otegui MS, Grotewold E 2014 Not all anthocyanins are born equal: distinct patterns induced by stress in Arabidopsis. Planta 240, 931–940. 24903357 10.1007/s00425-014-2079-1 PMC4200348

[CIT0028] Kroon J, Souer E, de Graaff A, Xue Y, Mol J, Koes R 1994 Cloning and structural analysis of the anthocyanin pigmentation locus *Rt* of *Petunia hybrida*: characterization of insertion sequences in two mutant alleles. The Plant Journal 5, 69–80. 8130799 10.1046/j.1365-313x.1994.5010069.x

[CIT0029] Kubo A, Arai Y, Nagashima S, Yoshikawa T 2004 Alteration of sugar donor specificities of plant glycosyltransferases by a single point mutation. Archives of Biochemistry and Biophysics 429, 198–203. 15313223 10.1016/j.abb.2004.06.021

[CIT0030] Kubo H, Nawa N, Lupsea SA 2007 *Anthocyaninless1* gene of *Arabidopsis thaliana* encodes a UDP-glucose:flavonoid-3-O-glucosyltransferase. Journal of Plant Research 120, 445–449. 17277900 10.1007/s10265-006-0067-7

[CIT0031] Kuromori T, Hirayama T, Kiyosue Y, Takabe H, Mizukado S, Sakurai T, Akiyama K, Kamiya A, Ito T, Shinozaki K 2004 A collection of 11 800 single-copy *Ds* transposon insertion lines in Arabidopsis. The Plant Journal 37, 897–905. 14996221 10.1111/j.1365.313x.2004.02009.x

[CIT0032] Lairson LL, Henrissat B, Davies GJ, Withers SG 2008 Glycosyltransferases: structures, functions, and mechanisms. Annual Review of Biochemistry 77, 521–555. 10.1146/annurev.biochem.76.061005.092322 18518825

[CIT0033] Leuzinger K, Dent M, Hurtado J, Stahnke J, Lai H, Zhou X, Chen Q 2013 Efficient agroinfiltration of plants for high-level transient expression of recombinant proteins. Journal of Visualized Experiments 77, 50521. 10.3791/50521 PMC3846102 23913006

[CIT0034] Lee MJ, Park JS, Choi DS, Jung MY 2013 Characterization and quantitation of anthocyanins in purple-fleshed sweet potatoes cultivated in Korea by HPLC-DAD and HPLC-ESI-QTOF-MS/MS. Journal of Agricultural and Food Chemistry 61, 3148–3158. 23464823 10.1021/jf3055455

[CIT0035] Mano H, Ogasawara F, Sato K, Higo H, Minobe Y 2007 Isolation of a regulatory gene of anthocyanin biosynthesis in tuberous roots of purple-fleshed sweet potato. Plant Physiology 143, 1252–1268. 17208956 10.1104/pp.106.094425 PMC1820918

[CIT0036] Matsuba Y, Sasaki N, Tera M, et al 2010 A novel glucosylation reaction on anthocyanins catalyzed by acyl-glucose-dependent glucosyltransferase in the petals of carnation and delphinium. The Plant Cell 22, 3374–3389. 20971893 10.1105/tpc.110.077487 PMC2990145

[CIT0037] Modolo LV, Li L, Pan H, Blount JW, Dixon RA, Wang X 2009 Crystal structures of glycosyltransferase UGT78G1 reveal the molecular basis for glycosylation and deglycosylation of (iso)flavonoids. Journal of Molecular Biology 392, 1292–1302. 19683002 10.1016/j.jmb.2009.08.017

[CIT0038] Moglia A, Lanteri S, Comino C, Hill L, Knevitt D, Cagliero C, Rubiolo P, Bornemann S, Martin C 2014 Dual catalytic activity of hydroxycinnamoyl-coenzyme A quinate transferase from tomato allows it to moonlight in the synthesis of both mono- and dicaffeoylquinic acids. Plant Physiology 166, 1777–1787. 25301886 10.1104/pp.114.251371 PMC4256858

[CIT0039] Montefiori M, Espley RV, Stevenson D, Cooney J, Datson PM, Saiz A, Atkinson RG, Hellens RP, Allan AC 2011 Identification and characterisation of F3GT1 and F3GGT1, two glycosyltransferases responsible for anthocyanin biosynthesis in red-fleshed kiwifruit (*Actinidia chinensis*). The Plant Journal 65, 106–118. 21175894 10.1111/j.1365-313X.2010.04409.x

[CIT0040] Morita Y, Hoshino A, Kikuchi Y, et al 2005 Japanese morning glory dusky mutants displaying reddish-brown or purplish-gray flowers are deficient in a novel glycosylation enzyme for anthocyanin biosynthesis, UDP-glucose:anthocyanidin 3-O-glucoside-2″-O-glucosyltransferase, due to 4-bp insertions in the gene. The Plant Journal 42, 353–363. 15842621 10.1111/j.1365-313X.2005.02383.x

[CIT0041] Nelson BK, Cai X, Nebenführ A 2007 A multicolored set of *in vivo* organelle markers for co-localization studies in Arabidopsis and other plants. The Plant Journal 51, 1126–1136. 17666025 10.1111/j.1365-313X.2007.03212.x

[CIT0042] Noguchi A, Horikawa M, Fukui Y, Fukuchi-Mizutani M, Iuchi-Okada A, Ishiguro M, Kiso Y, Nakayama T, Ono E 2009 Local differentiation of sugar donor specificity of flavonoid glycosyltransferase in Lamiales. The Plant Cell 21, 1556–1572. 19454730 10.1105/tpc.108.063826 PMC2700533

[CIT0043] Offen W, Martinez-Fleites C, Yang M, Kiat-Lim E, Davis BG, Tarling CA, Ford CM, Bowles DJ, Davies GJ 2006 Structure of a flavonoid glucosyltransferase reveals the basis for plant natural product modification. The EMBO Journal 25, 1396–1405. 16482224 10.1038/sj.emboj.7600970 PMC1422153

[CIT0044] Ono E, Fukuchi-Mizutani M, Nakamura N, Fukui Y, Yonekura-Sakakibara K, Yamaguchi M, Nakayama T, Tanaka T, Kusumi T, Tanaka Y 2006 Yellow flowers generated by expression of the aurone biosynthetic pathway. Proceedings of the National Academy of Sciences, USA 103, 11075–11080. 10.1073/pnas.0604246103 PMC1544175 16832053

[CIT0045] Ono E, Homma Y, Horikawa M, Kunikane-Doi S, Imai H, Takahashi S, Kawai Y, Ishiguro M, Fukui Y, Nakayama T 2010 Functional differentiation of the glycosyltransferases that contribute to the chemical diversity of bioactive flavonol glycosides in grapevines (*Vitis vinifera*). The Plant Cell 22, 2856–2871. 20693356 10.1105/tpc.110.074625 PMC2947185

[CIT0046] Osmani SA, Bak S, Møller BL 2009 Substrate specificity of plant UDP-dependent glycosyltransferases predicted from crystal structures and homology modeling. Phytochemistry 70, 325–347. 19217634 10.1016/j.phytochem.2008.12.009

[CIT0047] Pireyre M, Burow M 2015 Regulation of MYB and bHLH transcription factors: a glance at the protein level. Molecular Plant 8, 378–388. 25667003 10.1016/j.molp.2014.11.022

[CIT0048] Poustka F, Irani NG, Feller A, Lu Y, Pourcel L, Frame K, Grotewold E 2007 A trafficking pathway for anthocyanins overlaps with the endoplasmic reticulum-to-vacuole protein-sorting route in Arabidopsis and contributes to the formation of vacuolar inclusions. Plant Physiology 145, 1323–1335. 17921343 10.1104/pp.107.105064 PMC2151709

[CIT0049] Saito K, Yonekura-Sakakibara K, Nakabayashi R, Higashi Y, Yamazaki M, Tohge T, Fernie AR 2013 The flavonoid biosynthetic pathway in Arabidopsis: structural and genetic diversity. Plant Physiology and Biochemistry 72, 21–34. 23473981 10.1016/j.plaphy.2013.02.001

[CIT0050] Sasaki N, Nishizaki Y, Ozeki Y, Miyahara T 2014 The role of acyl-glucose in anthocyanin modifications. Molecules 19, 18747–18766. 25405291 10.3390/molecules191118747 PMC6271837

[CIT0051] Sawada S, Suzuki H, Ichimaida F, Yamaguchi MA, Iwashita T, Fukui Y, Hemmi H, Nishino T, Nakayama T 2005 UDP-glucuronic acid:anthocyanin glucuronosyltransferase from red daisy (*Bellis perennis*) flowers. Enzymology and phylogenetics of a novel glucuronosyltransferase involved in flower pigment biosynthesis. The Journal of Biological Chemistry 280, 899–906. 15509561 10.1074/jbc.M410537200

[CIT0052] Shao H, He X, Achnine L, Blount JW, Dixon RA, Wang X 2005 Crystal structures of a multifunctional triterpene/flavonoid glycosyltransferase from *Medicago truncatula*. The Plant Cell 17, 3141–3154. 16214900 10.1105/tpc.105.035055 PMC1276034

[CIT0053] Stamatakis A 2014 RAxML version 8: a tool for phylogenetic analysis and post-analysis of large phylogenies. Bioinformatics 30, 1312–1313. 24451623 10.1093/bioinformatics/btu033 PMC3998144

[CIT0054] Stracke R, Jahns O, Keck M, Tohge T, Niehaus K, Fernie AR, Weisshaar B 2010 Analysis of PRODUCTION OF FLAVONOL GLYCOSIDES-dependent flavonol glycoside accumulation in *Arabidopsis thaliana* plants reveals MYB11-, MYB12- and MYB111-independent flavonol glycoside accumulation. New Phytologist 188, 985–1000. 20731781 10.1111/j.1469-8137.2010.03421.x

[CIT0055] Sun W, Liang L, Meng X, Li Y, Gao F, Liu X, Wang S, Gao X, Wang L 2016 Biochemical and molecular characterization of a flavonoid 3-O-glycosyltransferase responsible for anthocyanins and flavonols biosynthesis in *Freesia hybrida*. Frontiers in Plant Science 7, 410. 27064818 10.3389/fpls.2016.00410 PMC4815329

[CIT0056] Sun Y, Li H, Huang JR 2012 Arabidopsis TT19 functions as a carrier to transport anthocyanin from the cytosol to tonoplasts. Molecular Plant 5, 387–400. 22201047 10.1093/mp/ssr110

[CIT0057] Tamura K, Stecher G, Peterson D, Filipski A, Kumar S 2013 MEGA6: molecular evolutionary genetics analysis version 6.0. Molecular Biology and Evolution 30, 2725–2729. 24132122 10.1093/molbev/mst197 PMC3840312

[CIT0058] Tian Q, Konczak I, Schwartz SJ 2005 Probing anthocyanin profiles in purple sweet potato cell line (*Ipomoea batatas* L. Cv. Ayamurasaki) by high-performance liquid chromatography and electrospray ionization tandem mass spectrometry. Journal of Agricultural and Food Chemistry 53, 6503–6509. 16076141 10.1021/jf050671m

[CIT0059] Tohge T, Nishiyama Y, Hirai MY, et al 2005 Functional genomics by integrated analysis of metabolome and transcriptome of Arabidopsis plants over-expressing an MYB transcription factor. The Plant Journal 42, 218–235. 15807784 10.1111/j.1365-313X.2005.02371.x

[CIT0060] Truong VD, Deighton N, Thompson RT, McFeeters RF, Dean LO, Pecota KV, Yencho GC 2010 Characterization of anthocyanins and anthocyanidins in purple-fleshed sweetpotatoes by HPLC-DAD/ESI-MS/MS. Journal of Agricultural and Food Chemistry 58, 404–410. 20017481 10.1021/jf902799a

[CIT0061] Wang H, Fan W, Li H, Yang J, Huang J, Zhang P 2013 Functional characterization of dihydroflavonol-4-reductase in anthocyanin biosynthesis of purple sweet potato underlies the direct evidence of anthocyanins function against abiotic stresses. PLoS ONE 8, e78484. 24223813 10.1371/journal.pone.0078484 PMC3817210

[CIT0062] Wetterhorn KM, Newmister SA, Caniza RK, Busman M, McCormick SP, Berthiller F, Adam G, Rayment I 2016 Crystal structure of Os79 (Os04g0206600) from *Oryza sativa*: a UDP-glucosyltransferase involved in the detoxification of deoxynivalenol. Biochemistry 55, 6175–6186. 27715009 10.1021/acs.biochem.6b00709

[CIT0063] Xu W, Dubos C, Lepiniec L 2015 Transcriptional control of flavonoid biosynthesis by MYB-bHLH-WDR complexes. Trends in Plant Science 20, 176–185. 25577424 10.1016/j.tplants.2014.12.001

[CIT0064] Yang J, Bi HP, Fan WJ, Zhang M, Wang HX, Zhang P 2011 Efficient embryogenic suspension culturing and rapid transformation of a range of elite genotypes of sweet potato (*Ipomoea batatas* [L.] Lam.). Plant Science 181, 701–711. 21958713 10.1016/j.plantsci.2011.01.005

[CIT0065] Yang JY, Yan RX, Roy A, Xu D, Poissonl J, Zhang Y 2015 The I-TASSER Suite: protein structure and function prediction. Nature Methods 12, 7–8. 25549265 10.1038/nmeth.3213 PMC4428668

[CIT0066] Yonekura-Sakakibara K, Fukushima A, Nakabayashi R, et al 2012 Two glycosyltransferases involved in anthocyanin modification delineated by transcriptome independent component analysis in *Arabidopsis thaliana*. The Plant Journal 69, 154–167. 21899608 10.1111/j.1365-313X.2011.04779.x PMC3507004

[CIT0067] Yonekura-Sakakibara K, Hanada K 2011 An evolutionary view of functional diversity in family 1 glycosyltransferases. The Plant Journal 66, 182–193. 21443631 10.1111/j.1365-313X.2011.04493.x

[CIT0068] Yonekura-Sakakibara K, Nakabayashi R, Sugawara S, Tohge T, Ito T, Koyanagi M, Kitajima M, Takayama H, Saito K 2014 A flavonoid 3-O-glucoside:2″-O-glucosyltransferase responsible for terminal modification of pollen-specific flavonols in *Arabidopsis thaliana*. The Plant Journal 79, 769–782. 24916675 10.1111/tpj.12580 PMC4282749

[CIT0069] Yonekura-Sakakibara K, Nakayama T, Yamazaki M, Saito K 2008 Modification and stabilization of anthocyanins. In: Winefield C, Davies K, Gould K. eds. Anthocyanins. New York: Springer, 169–190.

[CIT0070] Zhang Y, Butelli E, Martin C 2014 Engineering anthocyanin biosynthesis in plants. Current Opinion in Plant Biology 19, 81–90. 24907528 10.1016/j.pbi.2014.05.011

[CIT0071] Zhao J, Huhman D, Shadle G, He XZ, Sumner LW, Tang Y, Dixon RA 2011 MATE2 mediates vacuolar sequestration of flavonoid glycosides and glycoside malonates in *Medicago truncatula*. The Plant Cell 23, 1536–1555. 21467581 10.1105/tpc.110.080804 PMC3101557

[CIT0072] Zhao J 2015 Flavonoid transport mechanisms: how to go, and with whom. Trends in Plant Science 20, 576–585. 26205169 10.1016/j.tplants.2015.06.007

