# Supplementary material for: A novel glycosyltransferase catalyses the transfer of glucose to glucosylated anthocyanins in purple sweet potato
Source: J Exp Bot. 2018 Aug 17;69(22):5444–59. doi: 10.1093/jxb/ery305 (PMC6255700; doi:10.1093/jxb/ery305)
Supplement: Supplementary Figures S1-S10 and Tables S1-S2 [file ery305_suppl_supplementary-figures_tables.pdf]

SUPPLEMENTARY DATA

Article title: UDP-glucose:anthocyanidin 3-*O*-glucoside-2’’-*O*-glucosyltransferase catalyzes further glycosylation of anthocyanins in purple *Ipomoea batatas*

Authors: Hongxia Wang, Chengyuan Wang, Weijuan Fan, JunYang, Ingo Appelhagen, Yinliang Wu, Peng Zhang

The following Supporting Information is available for this article:

Fig. S1 UDP-glucose binding sites of Ib3GGT. (A) Chemical structure of UDP-glucose. (B, C) Close-up views of the interactions of uridine moiety (B), and diphosphate moiety. (D) Overall structure of Ib3GGT modeling structure. UDP-glucose is showed with ball-and-stick model and the protein structure is showed with cartoon; the helix/sheet/loops are showed in cyan/red/magenta, respectively.

Fig. S2 Structure alignment of UGT homologs. (A) All structures are showed with ribbon and superposed by Coot (Emsley *et al.*, 2010). The PDB numbers of these structures are 2ACW, 2C1Z, 2PQ6, 2VG8, 3HBF, 3WC4, 5GL5, 5NLM, 5TMB and 5U6M. (B) Ligands in superposed structures are showed with sticks.

Fig. S3 Sequence alignment of Ib3GGT with 2VG8 and 2VCH proteins using SWISS-MODEL and I-TASSER.

Fig. S4 Sugar-donor binding sites in Ib3GGT and At3GGT. (A, B, C, D) Close-up views of the interactions of UDP-glucose with Ib3GGT (A), UDP-glucose with At3GGT (B), UDP-xylose with Ib3GGT (C), and UDP-xylose with At3GGT (D). UDP-glucose and UDP-xylose are showed with stick-and-ball in magenta and red, respectively; side chain residues are showed with stick; the hydrogen bonds are indicated by dashed lines.

Fig. S5 Difference in binding affinity of sugar analogs. (A, B, C, D) Ball-and-stick model of UDP-galactose (A), UDP-glucose (B), UDP-arabinose (C), and UDP-xylose (D). They are showed in grey, magenta, pink, and yellow, respectively. Chemical structure of galactose, glucose, arabinose, and xylose are showed in the corresponding positions.

Fig. S6 Amino acid sequence comparison of GGT analogs with Ib3GGT. The Thr-138

site is boxed.

Fig. S7 Anthocyanin pigmentation and component profiles in seedlings of the *ugt79b1-2* mutant and *Ib3GGT*-overexpressing *ugt79b1-2* transgenic line.

Fig. S8 *Ib3GGT* promoter sequence showing the two G-box sites.

Fig. S9 Leaf and root phenotypes of field-grown wild-type, Ib3GGT-RNAi-2 and Ib3GGT-OE-2 plant lines.

Fig. S10 Analysis of anthocyanin compounds in wild-type and *At3GGT*-overexpressing sweet potato plants as determined by HPLC-MS.

Table S1 Anthocyanin compounds in sweet potato and Arabidopsis.

Table S2 List of primers for gene expression analysis in Arabidopsis and sweet potato plant lines.

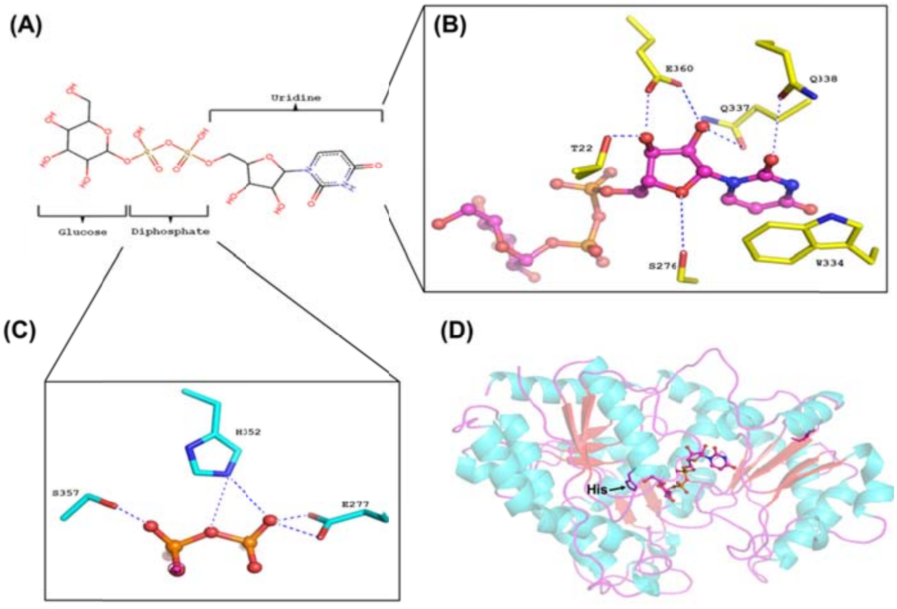

Fig. S1 UDP-glucose binding sites of Ib3GGT. (A) Chemical structure of UDP-glucose. (B, C) Close-up views of the interactions of uridine moiety (B), and diphosphate moiety. (D) Overall structure of Ib3GGT modeling structure. UDP-glucose is showed with ball-and-stick model and the protein structure is showed with cartoon; the helix/sheet/loops are showed in cyan/red/magenta, respectively.

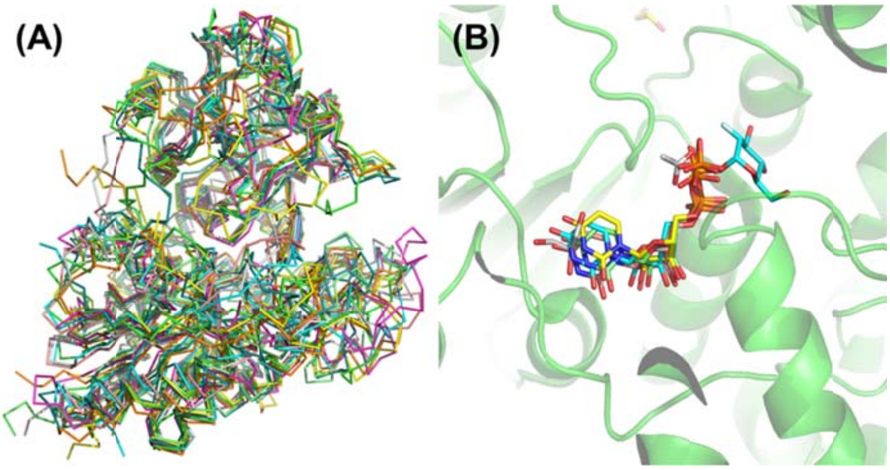

Fig. S2 Structure alignment of UGT homologs. (A) All structures are showed with ribbon and superposed by Coot (Emsley *et al.*, 2010). The PDB numbers of these structures are 2ACW, 2C1Z, 2PQ6, 2VG8, 3HBF, 3WC4, 5GL5, 5NLM, 5TMB and 5U6M. (B) Ligands in superposed structures are showed with sticks.

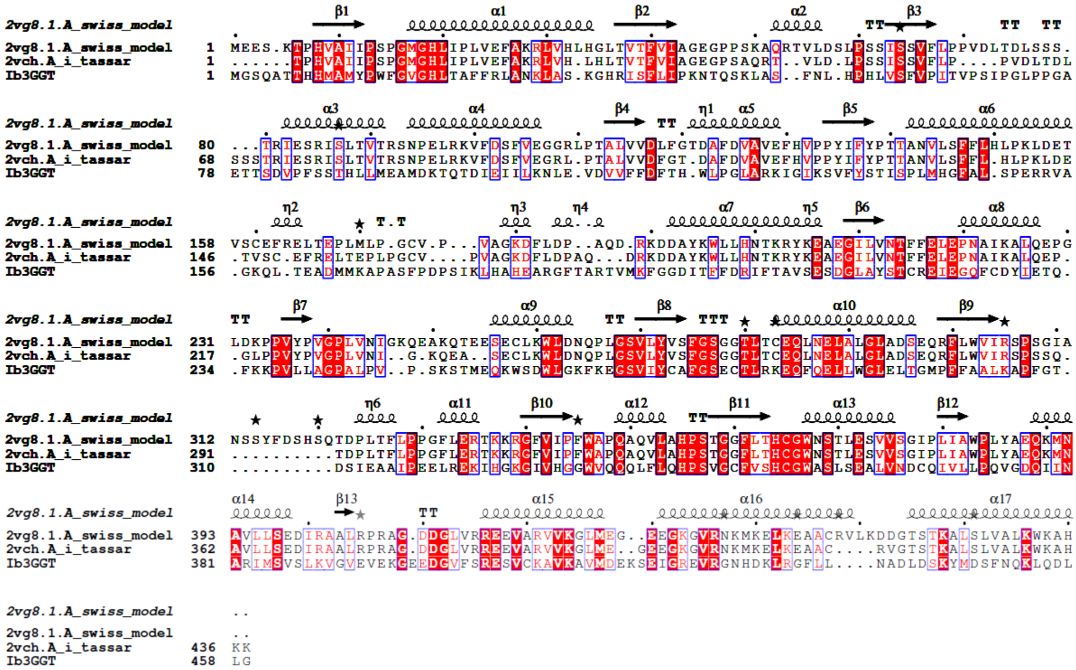

Fig. S3 Sequence alignment of Ib3GGT with 2VG8 and 2VCH proteins using SWISS-MODEL and I-TASSER.

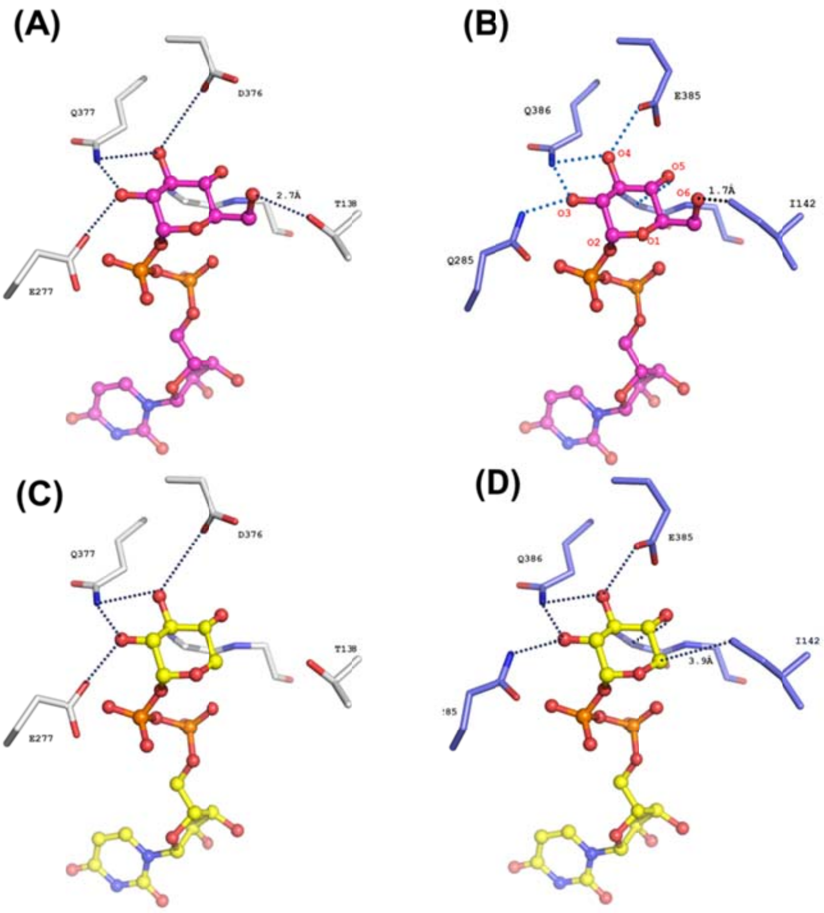

Fig. S4 Sugar-donor binding sites in Ib3GGT and At3GGT. (A, B, C, D) Close-up views of the interactions of UDP-glucose with Ib3GGT (A), UDP-glucose with At3GGT (B), UDP-xylose with Ib3GGT (C), and UDP-xylose with At3GGT (D). UDP-glucose and UDP-xylose are showed with stick-and-ball in magenta and red, respectively; side chain residues are showed with stick; the hydrogen bonds are indicated by dashed lines.

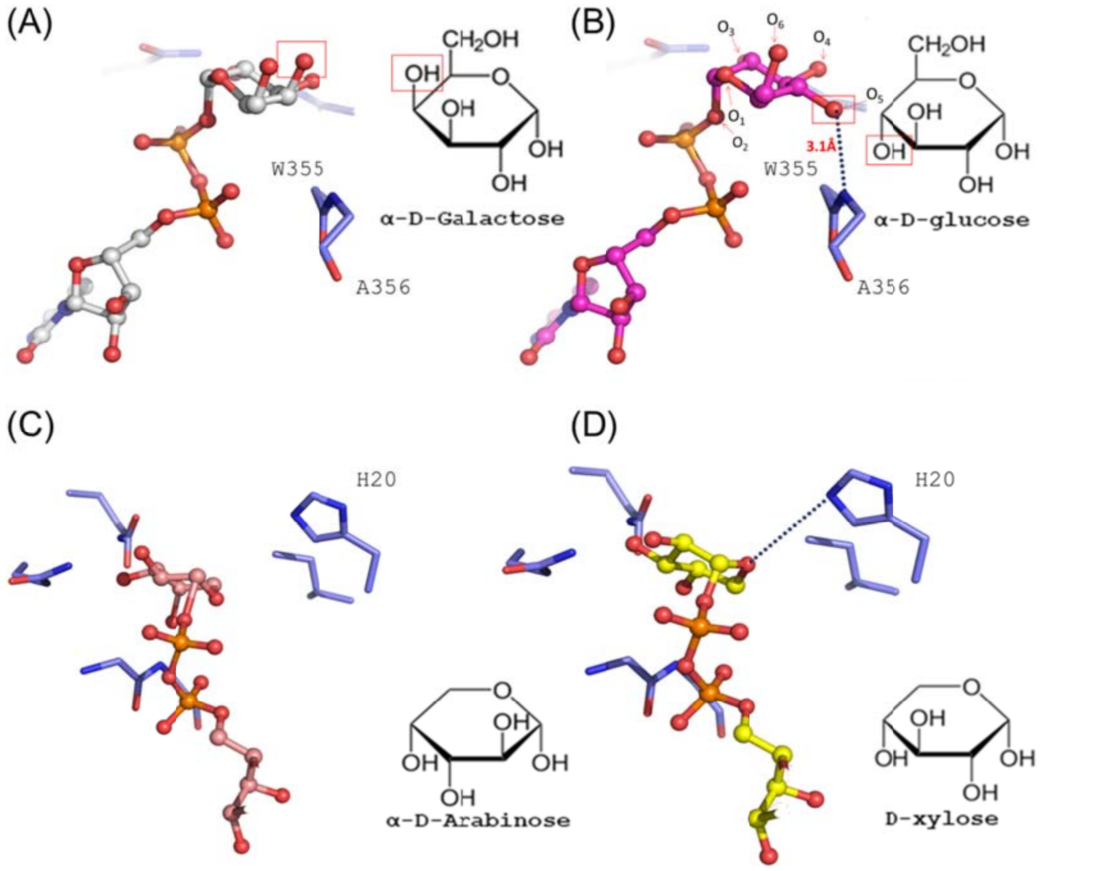

Fig. S5 Difference in binding affinity of sugar analogs. (A, B, C, D) Ball-and-stick model of UDP-galactose (A), UDP-glucose (B), UDP-arabinose (C), and UDP-xylose (D). They are showed in grey, magenta, pink, and yellow, respectively. Chemical structure of galactose, glucose, arabinose, and xylose are showed in the corresponding positions.

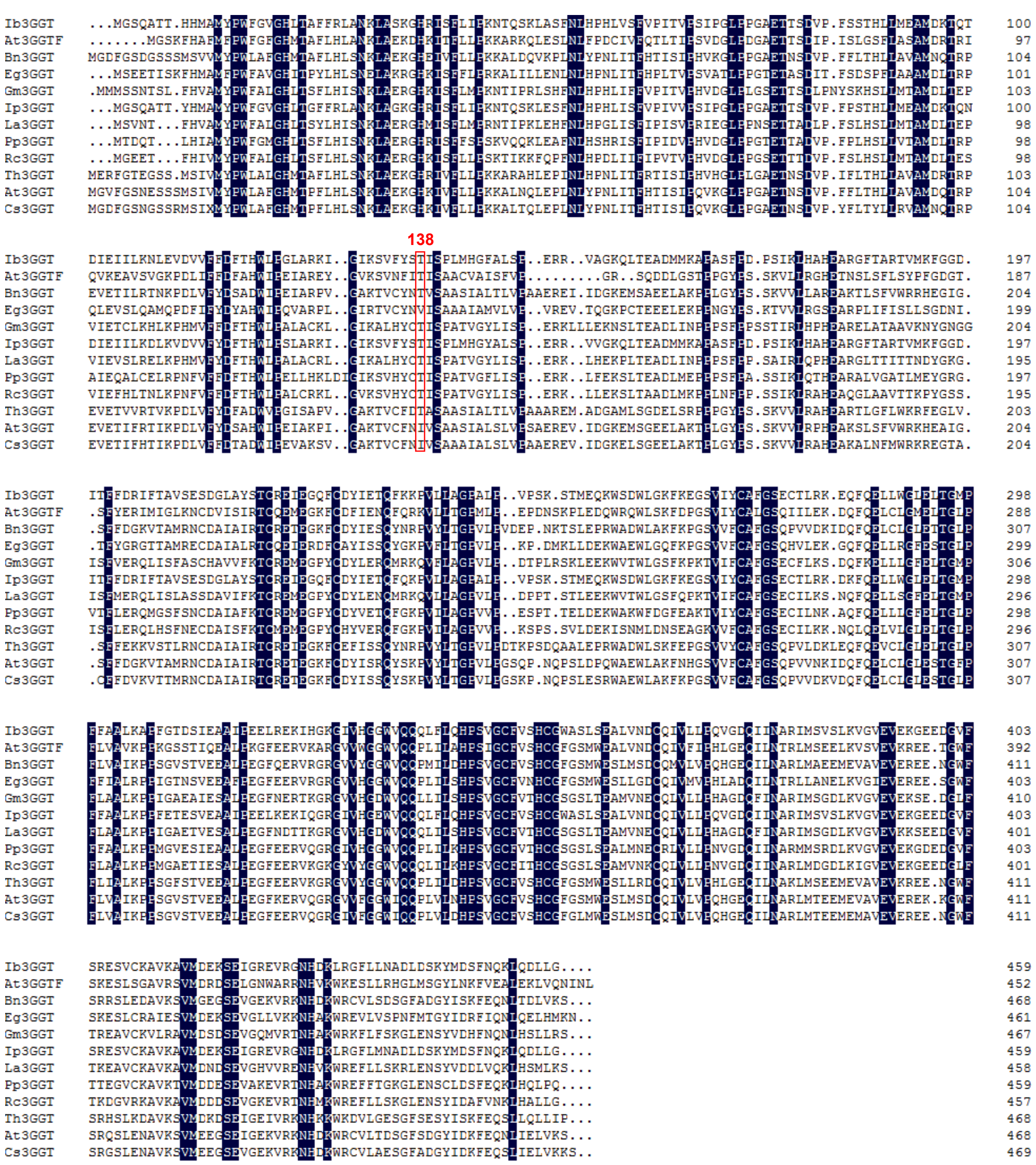

Fig. S6 Amino acid sequence comparison of different GGT analogs with Ib3GGT. The Thr-138 site is boxed.

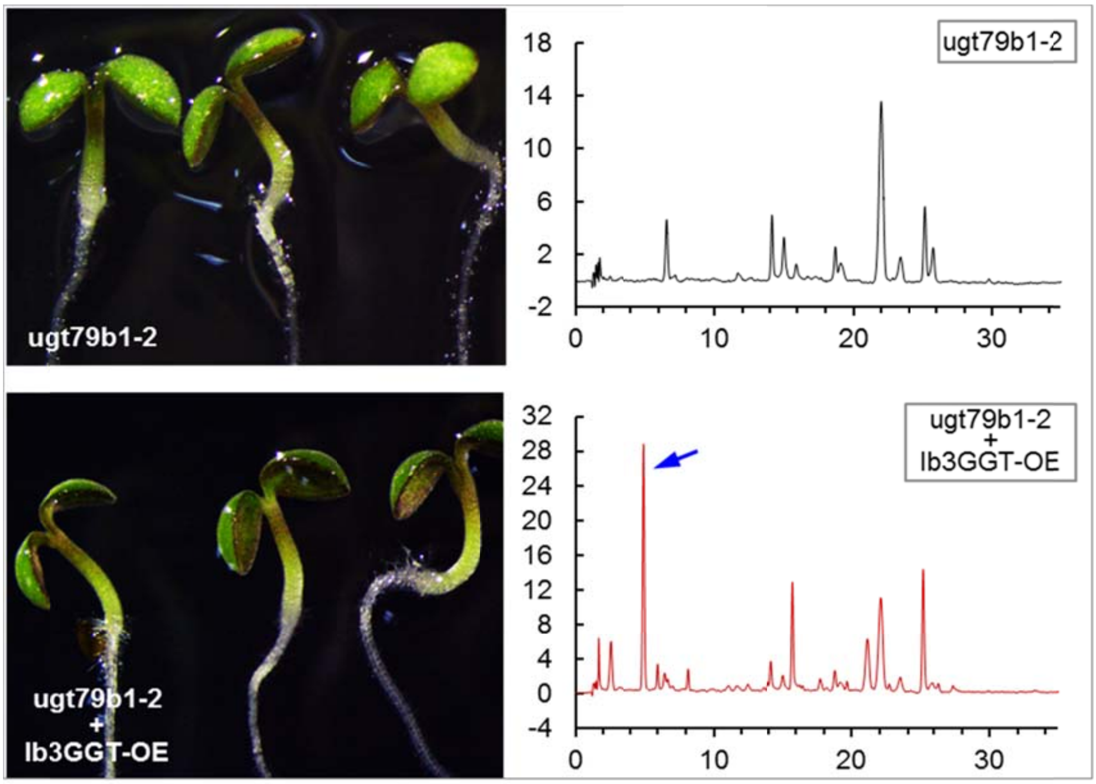

Fig. S7 Anthocyanin pigmentation and component profiles in seedlings of the *ugt79b1-2* mutant and *Ib3GGT*-overexpressing *ugt79b1-2* transgenic line. Blue arrows indicate the new cyanidin3-*O*-sophoroside.

Promoter of *Ib3GGT*

```
+ GCTGCCACAA TTAGGAAAGA GGTCGCAAAG CTTTGCAGTG AACTTCGACC ACACGCGCTT GCATTGGTGA
- CGACGGTGTT AATCCTTTCT CCAGCGTTTC GAAACGTCAC TTGAAGCTGG TGTGCGCGAA CGTAACCACT

+ ACTCCTTTGG CATACCTGAT GCTTTCTTGA GTCCCATAGC CTTTAATTGG ATAGATGCAA ATGCTTGGTC
- TGAGGAAACC GTATGGACTA CGAAAGAACT CAGGGTATCG GAAATTAACC TATCTACGTT TACGAACCAG

+ TTCCATTTAA GAATAATAGC TCATGACCTA CTGGAAAATT GAAGTGGTTG ACATCCATGG TTCTCTTCAG
- AAGGTAAATT CTTATTATCG AGTACTGGAT GACCTTTTAA CTTCACCAAC TGTAGGTACC AAGAGAAGTC

+ CAAATAAGAT GCAGAGAAGT TGTTCATCCC TCATCACATT CCTGCTTGTC ATCATCATCA AATAAAAGCT
- GTTTATTCTA CGTCTCTTCA ACAAGTAGGG AGTAGTGTAA GGACGAACAG TAGTAGTAGT TTATTTTCGA

+ ACTTTACTAT ATTATTACTT CTGTTAAAAC CTGTACTTGT TATAGTAAAA TGAATAAGGT AAATGTACCT
- TGAAATGATA TAATAATGAA GACAATTTTG GACATGAACA ATATCATTTT ACTTATTCCA TTTACATGGA

+ TTATTATTTA TAGCTATTCA CTATTTGTCT GGAAGTACAA ATAAATAATT AAAATAGTTA TTCATTCATC
- AATAATAAAT ATCGATAAGT GATAAACAGA CCTTCATGTT TATTTATTAA TTTTATCAAT AAGTAAGTAG

+ AGTGCTTCCT TTATGGCCCA CTCTTGCATC TCTTCTTTTG TTTTTTAGAA TGTTTTTGTT GATTAGTAAC
- TCACGAAGGA AATACCGGGT GAGAACGTAG AGAAGAAAAC AAAAAATCTT ACAAAAACAA CTAATCATTG
   G-box
+ CCACGTGGCA ACTCCTTCAC CTATAGCAAC TCTCACTAGC TGGGTTCAAA TTAACATTGA TAGTTGAAGG
- GGTGCACCGT TGAGGAAGTG GATATCGTTG AGAGTGATCG ACCCAAGTTT AATTGTAACT ATCAACTTCC

+ TTTATAAGAA AATTATAAAT AAGTTTGTAA TAATTATAAA ATTACTTCGT ATTTTTACAT GGGAGCTTGT
- AAATATTCTT TTAATATTTA TTCAAACATT ATTAATATTT TAATGAAGCA TAAAAATGTA CCCTCGAACA

+ TCTTACCTGA ACCTAACCCA TCACCTTCTA CACCTTTACG GTTCTATTCA ACCCTTCAAA TTCTTCTGAT
- AGAATGGACT TGGATTGGGT AGTGGAAGAT GTGGAAATGC CAAGATAAGT TGGGAAGTTT AAGAAGACTA
                                          G-box
+ CTGCTTTCAG TGGTAAAAGA ATGGATTCAT GCATGGTACG TGTTCTTTAA TTTTATGTGC GATGTAAACG
- GACGAAAGTC ACCATTTTCT TACCTAAGTA CGTACCATGC ACAAGAAATT AAAATACACG CTACATTTGC

+ TAACAGAGCC TACATGGTGA CATCATTGAC TGTGTTCAGA TCTCTCACCA ACCAGCTTTT GATCATCTCT
- ATTGTCTCGG ATGTACCACT GTAGTAACTG ACACAAGTCT AGAGAGTGGT TGGTCGAAAA CTAGTAGAGA

+ CAAGAATCGT GCAATTCAGG TAGCCTCAAC CCCCGGCCCA TCTCTTTTGT CTGTATATAC AAACACGATA
- GTTCTTAGCA CGTTAAGTCC ATCGGAGTTG GGGGCCGGGT AGAGAAAACA GACATATATG TTTGTGCTAT

+ TGGAAAAAGT TTGGCCATGC TTATGGTGGT TGTCACATGA TGAGCTCAAC TTACCAGTTT AAGAAGATAT
- ACCTTTTTCA AACCGGTACG AATACCACCA ACAGTGTACT ACTCGAGTTG AATGGTCAAA TTCTTCTATA

+ ACCTGCTCCA CCACAATCTT GCATTACACA TATTATTTGA TCCAAAACTG AAATAATATC AATTCCTATA
- TGGACGAGGT GGTGTTAGAA CGTAATGTGT ATAATAAACT AGGTTTTGAC TTTATTATAG TTAAGGATAT

+ TTTTGAACTT ACAAATGTTG GCCAAGTAAA AAAAAAAACT TCTTATTCTC AAGCTAATTA TCAAACCAAC
- AAAACTTGAA TGTTTACAAC CGGTTCATTT TTTTTTTTGA AGAATAAGAG TTCGATTAAT AGTTTGGTTG

+ TTAACTGAAG TTGCTCTCAG AAATAATCTC ATTCTAAACC TTATAGTCTT TAATATGTAA CAAAATTTGG
- AATTGACTTC AACGAGAGTC TTTATTAGAG TAAGATTTGG AATATCAGAA ATTATACATT GTTTTAAACC

+ GTGGTGTCAA TGGATAAATG TTATCCCTTA ATTGAATGGA TAAATGTTAG CAATTAAGTG TCCTAAGTGA
- CACCACAGTT ACCTATTTAC AATAGGGAAT TAACTTACCT ATTTACAATC GTTAATTCAC AGGATTCACT

+ CCACAAACAC CTCTTTTGGA CTGCATGTGC AACAGTCAGT CAGAGCGGGT TGGTTGAGAT TTGAGAAGTA
- GGTGTTTGTG GAGAAAACCT GACGTACACG TTGTCAGTCA GTCTCGCCCA ACCAACTCTA AACTCTTCAT

+ AAAAGCATTT AGAACAGCCG GGCAAGTGAG ATCAGAACAG CCCGGCGAGT GAAAACTTCA ATCAATAAAG
- TTTTCGTAAA TCTTGTCGGC CCGTTCACTC TAGTCTTGTC GGGCCGCTCA CTTTTGAAGT TAGTTATTTC

+ TAGCCAGTTG GCTATCATAT CTCTGCATGC ATAAAACATC TCACTCTCCA ATAATTCATC ACAATTGAAT
- ATCGGTCAAC CGATAGTATA GAGACGTACG TATTTTGTAG AGTGAGAGGT TATTAAGTAG TGTTAACTTA

+ TTGCAGAAAG CTAGCTAGCT AGGTATTAT  TATG
- AACGTCTTTC GATCGATCGA TCCATAATA  T └──→ Translation
```

Fig. S8 *Ib3GGT* promoter sequence showing the two G-box sites.

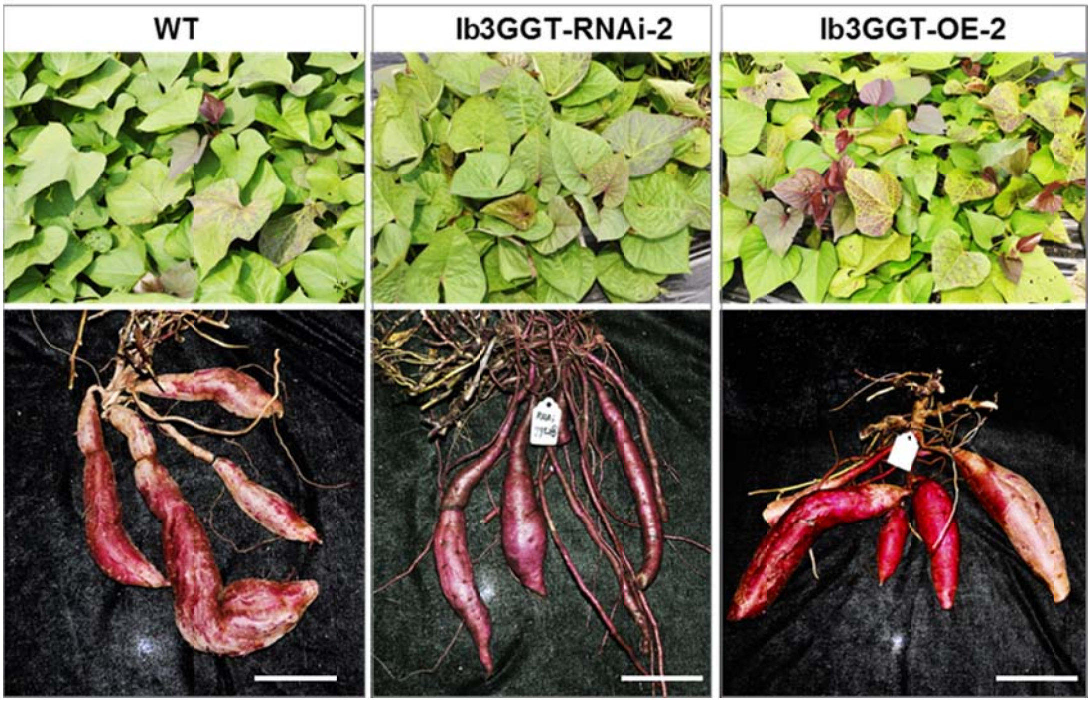

Fig. S9 Leaf and root phenotypes of field-grown WT, Ib3GGT-RNAi-2 and Ib3GGT-OE-2 plant lines.

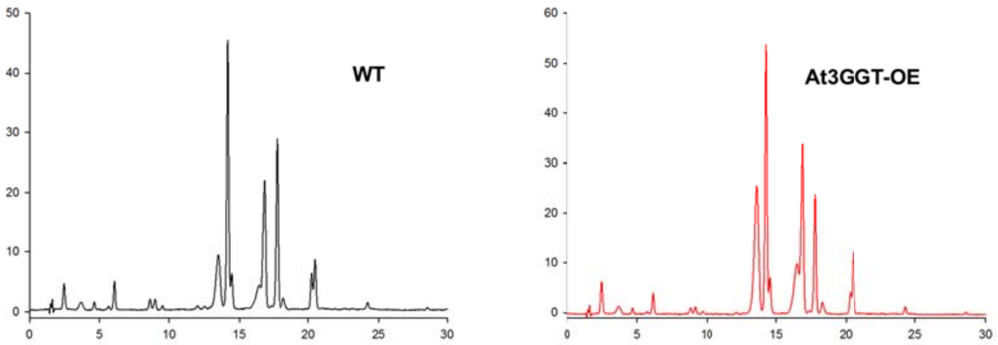

Fig. S10 Analysis of anthocyanin compounds in wild-type and *At3GGT*-overexpressing sweet potato plants as determined by HPLC-MS.

Table S1 Anthocyanin compounds in sweet potato and Arabidopsis.

	Sweet potato		Arabidopsis
1	Cyanidin 3-sophoroside-5-glucoside	A1	Cyanidin 3-*O*-[2”-*O*-(xylosyl) glucoside] 5-*O*-glucoside
2	Cyanidin 3,5-diglucoside	A2	Cyanidin 3-*O*-[2”-*O*-(xylosyl) glucoside] 5-*O*-(6”’-*O*-malonyl)glucoside
3	Pelargonidin 3-sophoroside-5-glucoside	A3	Cyanidin 3-*O*-[2”-*O*-(xylosyl)6”-*O*-(*p*-coumaroyl) glucoside] 5-*O*-glucoside
4	Peonidin 3-sophoroside-5-glucoside	A4	Cyanidin 3-*O*-[2”-*O*-(2”’-*O*-(sinapoyl)xylosyl) glucoside] 5-*O*-glucoside
5	Cyanidin 3-*p*-hydroxybenzoylsophoroside-5-glucoside	A5	Cyanidin 3-*O*-[2”-*O*-(xylosyl)-6”-*O*-(*p*-coumaroyl) glucoside]
6	Cyanidin 3-(6’’-caffeoylsophoroside)-5-glucoside		5-*O*-malonylglucoside
7	Peonidin 3-*p*-hydroxybenzoylsophoroside-5-glucoside	A6	Cyanidin 3-*O*-[2”-*O*-(xylosyl)-6”-*O*-(*p*-*O*-(glucosyl)-*p*-coumaroyl)glucoside]
8	Peonidin 3-caffeoylsophoroside-5-glucoside		5-*O*-glucoside
9	Cyanidin 3-(6”-*p*-coumarylsophoroside)-5-glucoside	A7	Cyanidin 3-*O*-[2”-*O*-(2”’-*O*-(sinapoyl)xylosyl)
10	Cyanidin 3-(6’’-feruloylsophoroside)-5-glucoside		6”-*O*-(*p*-coumaroyl)glucoside] 5-*O*-glucoside
11	Peonidin 3-(6″-*p*-coumarylsophoroside)-5-glucoside	A8	Cyanidin 3-*O*-[2”-*O*-(xylosyl) 6”-*O*-(*p*-*O*-(glucosyl) *p*-coumaroyl)glucoside]
12	Peonidin 3-(6’’-feruloylsophoroside)-5-glucoside		5-*O*-[6”’-*O*-(malony)glucoside]
13	Pelargonidin 3-feruloylsophoroside-5-glucoside	A9	Cyanidin3-*O*-[2”-*O*-(2”’-*O*-(sinapoyl)xylosyl)6”-*O*-(*p*-*O*-coumaroyl)
14	Unidentified		glucoside] 5-*O*-[6”’-*O*-(malony)glucoside]
15	Unidentified	A10	Cyanidin3-*O*-[2”-*O*-(2”’-*O*-(sinapoyl)xylosyl)6”-*O*-(*p*-*O*-(glucosyl)
16	Cyanidin 3-(6’’,6’’’-dicaffeoylsophoroside)-5-glucoside		*p*-coumaroyl) glucoside] 5-*O*-glucoside]
17	Cyanidin 3-caffeoylsophoroside-5-glucoside	A11	Cyanidin3-*O*-[2”-*O*-(6”’-*O*-(sinapoyl)xylosyl)6”-*O*-(*p*-*O*-(glucosyl)-
18	Cyanidin3-(6’’-caffeoyl-6’’’-feruloylsophoroside)-5-glucoside		*p*-coumaroyl) glucoside] 5-*O*-(6””-*O*-malonyl) glucoside
19	Cyanidin 3-caffeoyl-*p*-coumarylsophoroside-5-glucoside		
20	Peonidin 3-caffeoyl-*p*-hydroxybenzoyl-sophoroside-5-glucoside		
21	Peonidin 3-caffeoylsophoroside-5-glucoside		
22	Peonidin 3-feruloyl-*p*-caffeoylsophoroside-5-glucoside		
23	Cyanidin 3-feruloyl-*p*-coumarylsophoroside-5-glucoside		
24	Cyanidin 3-(6’’, 6’’’-dicoumarylsophoroside)-5-glucoside		
25	Peonidin 3-feruloyl-*p*-coumarylsophoroside-5-glucoside		
26	Peonidin 3-(6’’, 6’’’-dicoumarylsophoroside)-5-glucoside		

The data was based on sweet potato (Tian *et al.*, 2005) and Arabidopsis (Tohge *et al.*, 2005).

Table S2 List of primers for gene expression analysis in Arabidopsis and sweetpotato plant lines.

Primer pair	Forward primer (5'→3')	Reverse primer (5'→3')
**qIb3GGT**	CAGCAATTGTTTCTCCAGCA	TTCTTCCCCTTTCTCCACCT
**qIbActin**	TCTTGATCTTGCTGGTCGTG	TTTGGCTGTCTCGAGTTCCT
**qAt3GGT**	CCATTGGATACCGGAAATTG	CCACACGAAACTCAGGGATT
**qAtActin**	TCTTGATCTTGCTGGTCGTG	TTTGGCTGTCTCGAGTTCCT
